# Supplementary material for: The geometric nature of weights in real complex networks
Source: Nat Commun. 2017 Jan 18;8:14103. doi: 10.1038/ncomms14103 (PMC5253659; doi:10.1038/ncomms14103)
Supplement: Supplementary Information — Supplementary Figures, Supplementary Table 1, Supplementary Methods and Supplementary References. [file ncomms14103-s1.pdf]

Supplementary Methods

Further information and details about the model and the methods described in the main test are provided. Section I details the analytical results that can be obtained for the model presented in the paper. For the sake of completeness, some details discussed in the main text are reproduced. Section II discusses the method used to infer the value of α via the triangle inequality test. Section III compares the predictions of our model with other models proposed in the literature.

I. FURTHER DETAILS ON THE MODEL

Our model is a non-trivial generalization to weighted networks of a class of random networks with hidden variables embedded in a metric space [1]. In this model, N nodes are uniformly distributed with density δ in a D-dimensional homogeneous and isotropic metric space, and are assigned a hidden variable κ according to the probability density function (pdf) $\rho(\kappa)$. Two nodes with hidden variables κ and κ' separated by a metric distance d are connected with a probability

$$p(\kappa, \kappa', d) = p(\chi) \quad \text{where} \quad \chi = \frac{d}{(\mu\kappa\kappa')^{1/D}} \ , \tag{1}$$

where $\mu > 0$ is a free parameter and $p(\chi)$ is an arbitrary positive function taking values within the interval $(0, 1)$. Whenever the integral $\int_0^\infty \chi^{D-1} p(\chi) d\chi$ is bounded, the free parameter μ can be chosen such that $\bar{k}(\kappa) = \kappa$. Hence, κ corresponds to the expected degree of nodes, so the degree distribution can be specified through the pdf $\rho(\kappa)$

$$P(k) = \frac{1}{k!} \int e^{-\kappa} \kappa^k \rho(\kappa) d\kappa \ , \tag{2}$$

regardless of the specific form of $p(\chi)$ (see Secs. I A 1 and I A 2 below for details). The freedom in the choice of $p(\kappa, \kappa', d)$ allows us to tune the level of coupling between the topology of the networks and the metric space, which in turn allows us to control many properties such as the clustering coefficient and the navigability [1, 2]. Moreover, the form of the connection probability in Eq. (1) implies that networks generated with this model are small worlds for any heterogeneous pdf $\rho(\kappa)$ since high degree nodes are then likely to be connected regardless of the metric distance between them [1, 3].

To generalize this model to weighted networks, a second hidden variable σ is associated to each node. This new hidden variable can be correlated with κ so, hereafter, we assume that the pair of hidden variables (κ, σ) associated with the same node are drawn from the joint pdf $\rho(\kappa, \sigma)$. The weight of an *existing* link between two nodes with hidden variables κ, σ, κ' and σ', respectively, and at a metric distance d is distributed according to the pdf

$$\phi(w|\kappa, \sigma, \kappa', \sigma', d) = \frac{1}{\bar{w}} f\left(\frac{w}{\bar{w}}\right) \ , \tag{3}$$

where $f(\epsilon)$ is any probability density function in the domain $[0, \infty)$, and where

$$\bar{w} = \frac{\nu\sigma\sigma'}{(\kappa\kappa')^{1-\alpha/D} d^\alpha} \ , \tag{4}$$

with $\nu > 0$ and $0 \leq \alpha < D$. The particular form of the distribution of weights Eq. (3) implies that the weight between nodes i and j can be written as

$$\omega_{ij} = \epsilon_{ij} \frac{\nu\sigma_i\sigma_j}{(\kappa_i\kappa_j)^{1-\alpha/D} d_{ij}^\alpha} \tag{5}$$

where ϵ_{ij} is a random variable drawn from the pdf function $f(\cdot)$. Equations (3) and (4) constitute the keystone of our model. Indeed, as shown in Sec. I A 3 below, the form of Eq. (4) is the only ensuring that $\bar{s}(\sigma) = \sigma$, provided that the integral $\int_0^{\infty} \chi^{D-\alpha-1} p(\chi) d\chi$ converges. The new hidden variable σ can therefore be interpreted as the expected strength of a node, and the joint pdf $\rho(\kappa, \sigma) = \rho(\kappa)\rho(\sigma|\kappa)$ controls the correlation between degrees and strengths in the network. Indeed, as shown in Sec. I A 4 below, the average strength of nodes with a given degree, $\bar{s}(k)$, relates to the first moment of the conditional pdf $\rho(\sigma|\kappa)$, $\bar{\sigma}(\kappa)$, through the relation

$$\bar{s}(k) = \frac{1}{(k-1)!P(k)} \int \mathrm{e}^{-\kappa} \kappa^{k-1} \rho(\kappa) \bar{\sigma}(\kappa) d\kappa \ . \tag{6}$$

Therefore, when $\lim_{\kappa \to \infty} \bar{\sigma}(\kappa) = \infty$ then $\bar{s}(k) \sim \bar{\sigma}(\kappa)$. This limit stands as a good approximation for the behaviour of high degree nodes in real weighted networks.

Remarkably, these relations hold independently of the specific form of the connection probability $p(\chi)$ and of the distribution of weights $f(\epsilon)$, thus conferring great versatility to our model. Even more remarkable, the shape of the connection probability $p(\chi)$ and the value of the parameter α—coupling topology and weights to the metric space—do not affect the relations $\bar{k}(\kappa) = \kappa$ and $\bar{s}(\sigma) = \sigma$ and, therefore, the join degree-strength distribution $P(k, s)$. This property conveys a degree of control over the weight distribution as well as over the disparity of nodes which is independent of the specification of degrees and strengths and, more importantly, opens the possibility to measure the metric properties of complex weighted networks.

A. Theoretical calculations in a D-dimensional metric space

Most of the theoretical calculations for the model are carried out using the probability density function (pdf) $g(k, s|\kappa, \sigma)$ corresponding to the probability that a node with hidden variables κ and σ has a degree equal to k and a strength in the interval $[s, s + ds)$. To compute this pdf, we first consider a pair of nodes whose positions in space are $\boldsymbol{x_i}$ and $\boldsymbol{x_j}$ and whose hidden variables are κ_i, σ_i and κ_j, σ_j, respectively. The pdf for the weight w_{ij} between these two nodes ($w_{ij} = 0$ means that there is no link) is

$$\begin{aligned}\Phi(w_{ij}|\kappa_i, \sigma_i; \kappa_j, \sigma_j; d_{ij}) = {} & [1 - p(\kappa_i, \kappa_j, d_{ij})]\delta(w_{ij}) \\ & + p(\kappa_i, \kappa_j, d_{ij})\phi(w_{ij}|\kappa_i, \sigma_i, \kappa_j, \sigma_j, d_{ij})\Theta(w_{ij}) \ ,\end{aligned} \tag{7}$$

where $\delta(\cdot)$ is the Dirac delta function and $\Theta(\cdot)$ is the left-continuous Heaviside step function [i.e., $\Theta(0) = 0$]. Without loss of generality, we take the perspective of node i, place it at the origin of the coordinate system, and integrate over the possible values of the hidden variables of node j

$$\Phi(w_{ij}|\kappa_i, \sigma_i) = \iiint \frac{\rho(\kappa_j, \sigma_j)}{V_D} \Phi(w_{ij}|\kappa_i, \sigma_i; \kappa_j, \sigma_j; d_{ij}) d\boldsymbol{x_j} d\sigma_j d\kappa_j \ . \tag{8}$$

In this last equation, $1/V_D$ is the (uniform) pdf for the position of nodes in the metric space. This last expression does not depend on the position of node i due to the isotropy, homogeneity and large size ($N \gg 1$) of the metric space. Note that for mathematical convenience, we consider the metric space to be a D-dimensional sphere of radius R. However, the constraint of having constant density implies that the radius diverges in the thermodynamic limit and, thus, the metric space is equivalent to a D-dimensional Euclidean space. Because the weights $\{w_{ij}\}_{i,j=1,\ldots,N}$ are assigned independently in the model, the probability that a node has a degree k_i and a strength s_i is equal to the product of the contribution of each potential $N - 1$ links, given that the number of existing

links is equal to k_i and that their weights sum up to s_i

$$g(k_i,s_i|\kappa_i,\sigma_i) = \prod_j \left[\int \Phi(w_{ij}|\kappa_i,\sigma_i) dw_{ij} \right] \delta\left(k_i - \sum_l \Theta(w_{il}) \right) \delta\left(s_i - \sum_l w_{il} \right) . \tag{9}$$

Although it is not possible to further the calculation and obtain a closed form the the pdf $g(k,s|\kappa,\sigma)$, many useful results can be obtained using its generating function defined as

$$\hat{g}(x,y|\kappa_i,\sigma_i) = \sum_{k_i=0}^{N-1} \int_0^\infty g(k_i,s_i|\kappa_i,\sigma_i) x^{k_i} \mathrm{e}^{-s_i y} ds_i \ , \tag{10}$$

which, dropping the subscripts i and j, can be written as

$$\hat{g}(x,y|\kappa,\sigma) = \left[\iiint \frac{\rho(\kappa',\sigma')}{V_D} \left\{ [1 - p(\kappa,\kappa',d)] + p(\kappa,\kappa',d) x \int \phi(w|\kappa,\sigma,\kappa',\sigma',d) \mathrm{e}^{-wy} dw \right\} d\boldsymbol{x}' d\sigma' d\kappa' \right]^{N-1} . \tag{11}$$

1. The degree of nodes

From Eq. (11), we can readily see that the average degree of nodes with hidden variables κ and σ only depends on κ [4]

$$\begin{aligned} \bar{k}(\kappa,\sigma) &= \left. \frac{\partial \hat{g}(x,y|\kappa,\sigma)}{\partial x} \right|_{x=1,y=0} \\ &= \frac{N-1}{V_D} \iiint \rho(\kappa',\sigma') p(\kappa,\kappa',d) d\boldsymbol{x}' d\sigma' d\kappa' \\ &= \delta \iint \rho(\kappa') p(\kappa,\kappa',d) d\boldsymbol{x}' d\kappa' \ , \end{aligned} \tag{12}$$

where $\rho(\kappa)$ is the marginal pdf of $\rho(\kappa,\sigma)$, and where $\delta \equiv N/V_D$ is the density of nodes in the metric space (we consider here that $N \gg 1$). Using the definition of $p(\kappa,\kappa',d)$ given in Eq. (1) and switching to D-dimensional spherical coordinates (Ω_D is the solid angle subtended by a D-dimensional object), this last equation becomes

$$\begin{aligned} \bar{k}(\kappa) &= \delta \iiint \rho(\kappa') p(\chi) r^{D-1} dr d\Omega_D d\kappa' \\ &= \frac{2\pi^{D/2}\delta\mu}{\Gamma(D/2)} \kappa \int \kappa' \rho(\kappa') d\kappa' \int \chi^{D-1} p(\chi) d\chi \\ &= \frac{2\pi^{D/2}\delta\mu\langle\kappa\rangle I_1}{\Gamma(D/2)} \kappa \ , \end{aligned} \tag{13}$$

where we have noted $\langle\kappa\rangle = \int \kappa\rho(\kappa)d\kappa$ and $I_1 = \int \chi^{D-1}p(\chi)d\chi$. The average degree for the whole network is

$$\langle k \rangle = \int \bar{k}(\kappa)\rho(\kappa)d\kappa = \frac{2\pi^{D/2}\delta\mu I_1}{\Gamma(D/2)} \langle\kappa\rangle^2 \ . \tag{14}$$

Consequently, we see that the free parameter μ can be chosen such that $\bar{k}(\kappa) = \kappa$ and $\langle k \rangle = \langle \kappa \rangle$, that is

$$\mu = \frac{\Gamma(D/2)}{2\pi^{D/2}\delta I_1 \langle \kappa \rangle} \ . \tag{15}$$

The degree distribution of the networks generated by the model can therefore be controlled through the pdf $\rho(\kappa)$. Following similar steps, we also find that

$$\mathrm{Var}[k(\kappa)] = \bar{k}(\kappa) \ , \tag{16}$$

which implies that

$$\frac{\sqrt{\mathrm{Var}[k(\kappa)]}}{\bar{k}(\kappa)} = \frac{1}{\sqrt{\kappa}} \ , \tag{17}$$

where we used $\bar{k}(\kappa) = \kappa$. In other words, nodes with a same high value of their hidden variable κ tend to all have a degree close to the expected value $\bar{k}(\kappa) = \kappa$.

2. The degree distribution

By definition, evaluating Eq. (11) at $y = 0$ yields the generating function for the degree distribution of nodes with hidden variable κ

$$\hat{g}(x, 0|\kappa, \sigma) \equiv \sum_k g(k|\kappa) x^k \ . \tag{18}$$

Assuming $N \gg 1$ and using Eq. (12), we obtain

$$\begin{aligned} \hat{g}(x, 0|\kappa, \sigma) &= \left[1 + (x-1)\frac{\bar{k}(\kappa)}{N-1} \right]^{N-1} \\ &= \exp\left\{ (x-1)\bar{k}(\kappa) \right\} \\ &= \sum_k \frac{\mathrm{e}^{-\bar{k}(\kappa)}\bar{k}(\kappa)^k}{k!} x^k \ . \end{aligned} \tag{19}$$

In other words, the degrees of nodes with hidden variable κ follow a Poisson distribution with mean $\bar{k}(\kappa)$ [4]. Using $\bar{k}(\kappa) = \kappa$, the degree distribution of the whole network is therefore

$$\begin{aligned} P(k) &= \int g(k|\kappa)\rho(\kappa) d\kappa \\ &= \frac{1}{k!} \int \mathrm{e}^{-\kappa} \kappa^k \rho(\kappa) d\kappa \ , \end{aligned} \tag{20}$$

thus unveiling the precise link between $\rho(\kappa)$ and $P(k)$.

3. The strength of nodes

Following similar steps as in Sec. I A 1 above, we can compute the average strength of nodes with hidden variables κ and σ

$$\begin{aligned}\bar{s}(\kappa,\sigma) &= -\left.\frac{\partial\hat{g}(x,y|\kappa,\sigma)}{\partial y}\right|_{x=1,y=0} \\ &= \delta \iiint \rho(\kappa',\sigma')p(\kappa,\kappa',d)\int w\phi(w|\sigma,\sigma',d)dw d\boldsymbol{x}' d\sigma' d\kappa' \\ &= \frac{2\pi^{D/2}\delta\mu^{1-\alpha/D}\nu I_2}{\Gamma(D/2)}\sigma\int\sigma'\rho(\sigma')d\sigma'\int\chi^{D-\alpha-1}p(\chi)d\chi \\ &= \frac{2\pi^{D/2}\delta\mu^{1-\alpha/D}\nu I_2\langle\sigma\rangle I_3}{\Gamma(D/2)}\sigma\ , \end{aligned} \tag{21}$$

where $\rho(\sigma)$ is the other marginal pdf of $\rho(\kappa,\sigma)$, and where we have noted $\langle\sigma\rangle = \int \sigma\rho(\sigma)d\sigma$, $I_2 = \int wf(w)dw$, and $I_3 = \int \chi^{D-\alpha-1}p(\chi)d\chi$. We therefore conclude that the strength of nodes only depends on σ, hence $\bar{s}(\kappa,\sigma) = \bar{s}(\sigma)$. The average strength for the whole network is

$$\begin{aligned}\langle s\rangle &= \int \bar{s}(\sigma)\rho(\sigma)d\sigma \\ &= \frac{2\pi^{D/2}\delta\mu^{1-\alpha/D}\nu I_2 I_3}{\Gamma(D/2)}\langle\sigma\rangle^2\ . \end{aligned} \tag{22}$$

As for the average degree, we see that we can set the free parameter ν such that $\bar{s}(\sigma) = \sigma$ and $\langle s\rangle = \langle\sigma\rangle$, that is

$$\nu = \frac{\Gamma(D/2)}{2\pi^{D/2}\delta\mu^{1-\alpha/D}I_2I_3\langle\sigma\rangle}\ . \tag{23}$$

The strength distribution of the networks generated by the model can therefore be controlled through the pdf $\rho(\sigma)$. Following similar steps, we find that

$$\begin{aligned}\mathrm{Var}[s(\kappa,\sigma)] &= \delta\iiint\rho(\kappa',\sigma')p(\kappa,\kappa',d)\int w^2\phi(w|\sigma,\sigma',d)dw d\boldsymbol{x}' d\sigma' d\kappa' \\ &= \frac{2\pi^{D/2}\delta\mu^{1-2\alpha/D}\nu^2 I_4\langle\sigma^2/\kappa\rangle I_5}{\Gamma(D/2)}\frac{\sigma^2}{\kappa}\ , \end{aligned} \tag{24}$$

where we have noted $\langle\sigma^2/\kappa\rangle = \iint(\sigma^2/\kappa)\rho(\kappa,\sigma)d\sigma d\kappa$, $I_4 = \int w^2 f(w)dw$ and $I_5 = \int\chi^{D-2\alpha-1}p(\chi)d\chi$. Setting μ such that $\langle k\rangle = \langle\kappa\rangle$ [see Eq. (14)], we find that

$$\frac{\sqrt{\mathrm{Var}[s(\kappa,\sigma)]}}{\bar{s}(\sigma)} = \frac{\sqrt{\bar{\kappa}\langle\sigma^2/\kappa\rangle I_1 I_4 I_5}}{\langle\sigma\rangle I_2 I_3}\frac{1}{\sqrt{\kappa}}\ , \tag{25}$$

i.e., the strength of high-degree nodes is close to its expected value given by Eq. (21).

4. The strength of nodes of degree k

Unfortunately, it is not possible to obtain a general closed form of the pdf $g(s|\kappa,\sigma)$, similar to Eq. (19), from Eq. (11). We can however characterize the strengths of nodes through the average

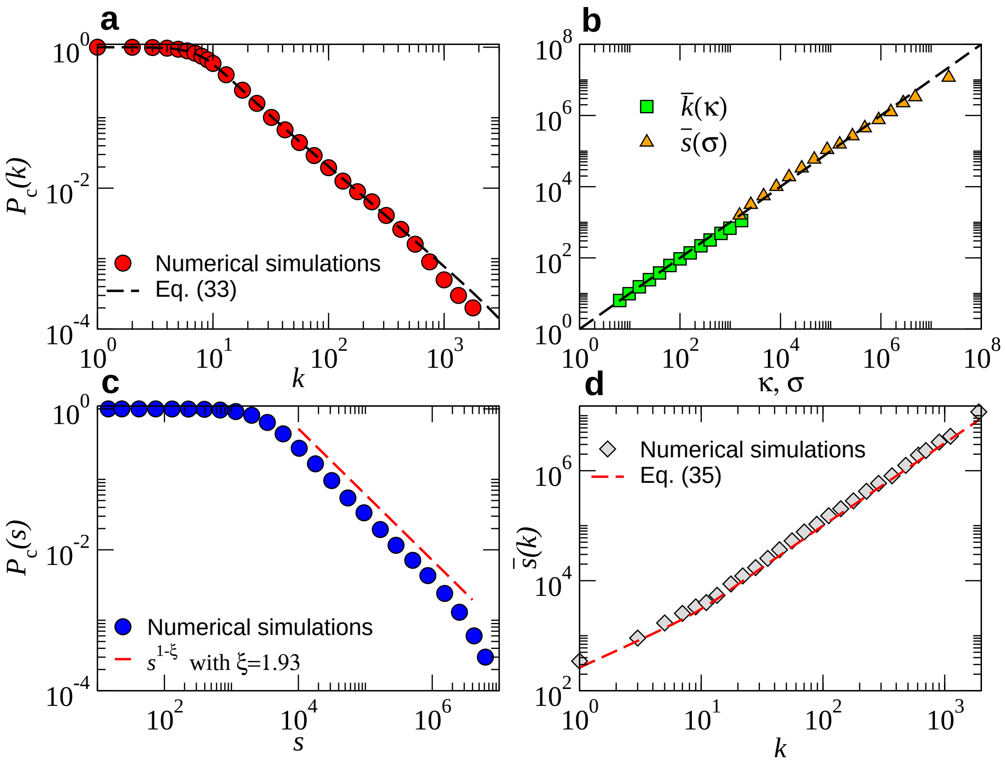

SUPPLEMENTARY FIGURE 1. **Validation of the theoretical calculations** The theoretical calculations are given in Sec. I B, and the numerical simulations correspond to a single network of size $N = 10^4$ and parameters $\alpha = 0.4$, $\beta = 1.5$, $\gamma = 2.4$, $\eta = 1.5$, $\langle k \rangle = 20$, $\kappa_c = \kappa_0 N^{1/(\gamma-1)}$, $a = 100$, and noise $\langle \epsilon^2 \rangle = 1.5$. **a**, complementary cumulative degree distribution compared with the prediction given by Eq. (33). **b**, average degree and average strength of nodes as a function of their hidden variables κ and σ. The deviation for high κ and σ is due to the finite size of the network and disappears as $N \to \infty$. **c**, complementary cumulative strength distribution. The dashed line indicates a scaling $\propto s^{-\xi}$ with $\xi = (\gamma + \eta - 1)/\eta \simeq 1.93$, as expected. **d**, average strength as a function of degree. The dashed line shows the prediction of Eq. (35).

strength of nodes with a given degree, $\bar{s}(k)$. Let us first explicit its calculation

$$\begin{aligned} \bar{s}(k) &= \int s g(s|k) ds \\ &= \frac{1}{P(k)} \int s g(k,s) ds \\ &= \frac{1}{P(k)} \iint \left[\int s g(k,s|\kappa,\sigma) ds \right] \rho(\kappa,\sigma) d\sigma d\kappa \ . \end{aligned} \tag{26}$$

From Eq. (10), we see that the integral in brackets can be obtained from Eq. (11)

$$-\left. \frac{\hat{g}(x,y|\kappa,\sigma)}{\partial y} \right|_{y=0} = \sum_k \left[\int s g(k,s|\kappa,\sigma) ds \right] x^k \ . \tag{27}$$

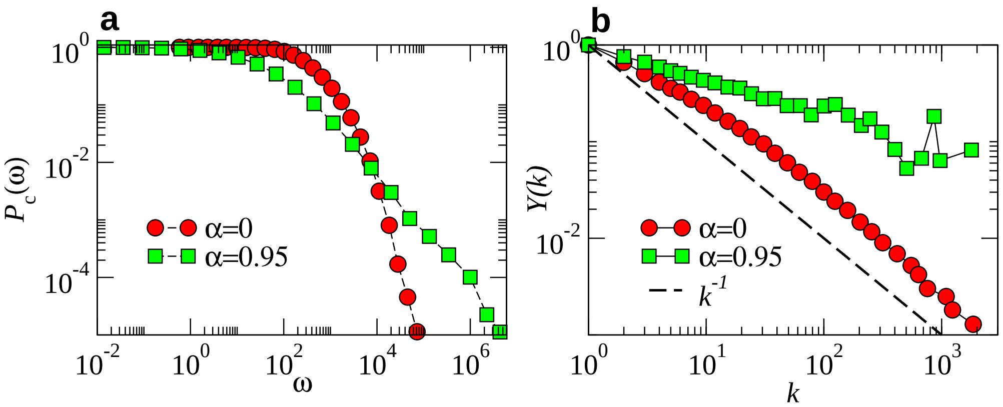

SUPPLEMENTARY FIGURE 2. **Effect of the underlying geometry on the weights** Simulations correspond to synthetic networks with the same parameters as in Supplementary Figure 1 but with two different values of the coupling parameter α. **a**, complementary cumulative weight distribution for $\alpha = 0$ (no coupling with the hidden metric space) and $\alpha = 0.95$ (strong coupling). **b**, average disparity of nodes as a function of their degree for the same values of the coupling α. The dashed line shows the scaling k^{-1}, corresponding to the a perfect equipartition of the strength of a node among its links.

Assuming $N \gg 1$ and using Eqs. (13) and (21), we find

$$\begin{aligned} -\left.\frac{\hat{g}(x,y|\kappa,\sigma)}{\partial y}\right|_{y=0} &= x\bar{s}(\sigma)\left[1+(x-1)\frac{\bar{k}(\kappa)}{N-1}\right]^{N-2} \\ &= x\bar{s}(\sigma)\mathrm{exp}\left\{(x-1)\bar{k}(\kappa)\right\} \\ &= \sum_{k>0}\left[\frac{\bar{s}(\sigma)\mathrm{e}^{-\bar{k}(\kappa)}\bar{k}(\kappa)^{k-1}}{(k-1)!}\right]x^k \ . \end{aligned} \tag{28}$$

Excluding the case $k = 0$ in this last expression is not problematic since $g(s|0) = \delta(s)$, by definition, and therefore the coefficient in front of x^0 must be zero [see Eq. (27)]. Combining Eqs. (26)–(28), $\bar{k}(\kappa) = \kappa$ and $\bar{s}(\sigma) = \sigma$, we obtain

$$\begin{aligned} \bar{s}(k) &= \frac{1}{(k-1)!P(k)}\iint \sigma \mathrm{e}^{-\kappa}\kappa^{k-1}\rho(\kappa,\sigma)d\sigma d\kappa \\ &= \frac{1}{(k-1)!P(k)}\int \mathrm{e}^{-\kappa}\kappa^{k-1}\rho(\kappa)\bar{\sigma}(\kappa)d\kappa \ . \end{aligned} \tag{29}$$

thus further clarifying how the joint pdf $\rho(\kappa, \sigma)$ controls the correlation between the degree and the strength of nodes. In fact, assuming no correlation, i.e., $\rho(\kappa, \sigma) = \rho(\kappa)\rho(\sigma)$, yields $\bar{s}(k) = \langle\sigma\rangle$, which is independent of the degree, as expected.

B. Validation of the theoretical calculations

All results presented in the previous section hold in arbitrary dimension and for any form of the connection probability $p(\chi)$ and weight probability density $f(\epsilon)$ in Eqs. (1) and (3). To validate the theoretical calculations, we particularize to the $\mathbb{S}^1$ model as generator of the topology [1]. In that model, we choose the circle $\mathbb{S}^1$ of radius $R = N/2\pi$ to be the underlying geometry over which nodes are uniformly distributed with density $\delta = 1$. Distances among nodes are measured in terms of arc lengths, that is, two nodes with angular positions θ and θ' are therefore at a distance

$d(\theta,\theta') = R\Delta\theta$ where $\Delta\theta = \pi - |\pi - |\theta - \theta'||$. The connection probability is set to

$$p(\chi) = \frac{1}{1+\chi^{\beta}} \quad \text{with } \chi = \frac{d}{\mu\kappa\kappa'}, \tag{30}$$

where $\beta > 1$ is a free parameter that can be used to tune the clustering. Equation (30) casts the ensemble of networks generated by the model into exponential random networks [5], i.e., networks that are maximally random given the constraints imposed by the free parameters (that is, $\rho(\kappa)$ and β). To obtain a scale-free degree distribution, hidden variables κ are distributed according to

$$\rho(\kappa) = \frac{(\gamma-1)\kappa_0^{\gamma-1}\kappa^{-\gamma}}{1-(\kappa_{\rm c}/\kappa_0)^{1-\gamma}} \tag{31}$$

with $\kappa_0 < \kappa < \kappa_{\rm c}$ and $\gamma > 1$. Notice that by keeping the explicit dependence in the upper cut-off it is possible to model networks with $\gamma < 2$ and a hard cut-off, as found for instance in airports networks [6]. Moreover, since it is generally more convenient to fix the average degree $\langle k\rangle$ explicitly, we choose κ_0 such that

$$\langle\kappa\rangle = \int_{\kappa_0}^{\kappa_{\rm c}} \kappa\rho(\kappa)d\kappa = \frac{(\gamma-1)\kappa_0}{(\gamma-2)}\frac{1-(\kappa_{\rm c}/\kappa_0)^{2-\gamma}}{1-(\kappa_{\rm c}/\kappa_0)^{1-\gamma}} = \langle k\rangle\ , \tag{32}$$

and fix the remaining free parameters $\kappa_{\rm c}$ and γ externally. From Eq. (2), we expect

$$P(k) = \frac{(\gamma-1)\kappa_0^{\gamma-1}}{1-(\kappa_{\rm c}/\kappa_0)^{1-\gamma}}\frac{\Gamma(k-\gamma+1,\kappa_0,\kappa_{\rm c})}{k!} \sim \frac{(\gamma-1)\kappa_0^{\gamma-1}k^{-\gamma}}{1-(\kappa_{\rm c}/\kappa_0)^{1-\gamma}} \sim k^{-\gamma}\ , \tag{33}$$

where $\Gamma(x,\kappa_0,\kappa_{\rm c})$ is the generalized incomplete gamma function. It is defined as $\Gamma(t,z_0,z_1) = \int_{z_0}^{z_1} z^{t-1}{\rm e}^{-z}dz$ (the regular complete and incomplete gamma functions can be retrieved by setting the bounds z_0 and z_1 to the appropriate values). Whenever $1 \sim z_0 \ll z_1$, a condition holding for most realistic degree distributions, the double incomplete gamma function scales as $\Gamma(n+\epsilon,\kappa_0,\kappa_{\rm c}) \sim \Gamma(n)n^{\epsilon}$ with $n \in \mathbb{N}$ and $\epsilon \in \mathbb{R}$ [7]. Note also that it is common to set $\kappa_{\rm c} = \kappa_0 N^{1/(\gamma-1)}$, i.e., the *natural* cut-off of a scale-free distribution [8, 9]. However, in general $\kappa_{\rm c}$ can take any value in response to particular mechanisms at play, like the limited capacity to handle more than a given number of connections in the airports network.

To assign weights on top of the topology generated by the model, the noise distribution in Eq. (3) is chosen to be a gamma distribution of average $\langle\epsilon\rangle = 1$, that is,

$$f(\epsilon) = \frac{\lambda^{\lambda}}{\Gamma(\lambda)}\epsilon^{\lambda-1}e^{-\lambda\epsilon} \quad \text{with } \langle\epsilon^2\rangle = 1+\frac{1}{\lambda}. \tag{34}$$

This particular choice allows us to interpolate with a single parameter between a zero noise limit when $\lambda \gg 1$, exponential noise when $\lambda = 1$, and strongly heterogeneous noise when $\lambda \ll 1$. Finally, to control the correlation between strength and degree and, therefore, to tune the strength distribution, we assume a deterministic relation between hidden variables σ and κ of the form $\sigma = a\kappa^{\eta}$, as observed in real complex networks [6]. From Eq. (6), we thus expect

$$\bar{s}(k) = \frac{ak\Gamma(k-\gamma+\eta,\kappa_0,\kappa_{\rm c})}{\Gamma(k-\gamma+1,\kappa_0,\kappa_{\rm c})} \sim ak^{\eta}\ . \tag{35}$$

Notice that the relation between average strength and degree in the previous expression is totally independent of the underlying metric space. It implies that the strength distribution scales as $P(s) \sim s^{-\xi}$ for $s \gg 1$ with $\xi = (\gamma+\eta-1)/\eta$. Supplementary Figure 1 shows the basic topological and weighted properties of a network generated using Eqs. (30)–(35) and compares them to the theoretical predictions presented in this section. Apart from some expected fluctuations due to finite size, the agreement between the two is excellent.

C. The effect of the underlying geometry

Geometry has a strong effect on the strength and weight distributions, which depend on the coupling parameter α. In fact, as shown in Sec. I A 3, the second moment of the strength distribution $\langle s^2 \rangle$ is proportional to the integral $\int_0^\infty \chi^{D-2\alpha-1} p(\chi) d\chi$, which diverges whenever $\alpha > D/2$. The origin of these fluctuations is rooted in the strong constraints that geometry imposes on the weights of individual links. In the absence of coupling (i.e., $\alpha = 0.0$) the metric distance between nodes does not influence the magnitudes of the weights. Consequently, the distribution of weights generated by the model is the original pdf in Eqs. (3) and (4) convoluted with the distribution of values of the ratio $\sigma\sigma'/\kappa\kappa'$. Conversely, in the case of strong coupling (i.e., $\alpha \lesssim 1$), short range links are constrained to have larger weights whereas long range ones have small weights. This effect increases the heterogeneity in the weight distribution and causes the divergence of $\langle s^2 \rangle$ when $\alpha > D/2$. Supplementary Figure 2**a** shows this effect on synthetic networks generated with the model with identical parameters except for the value of α.

The same effect is visible in the local heterogeneity of the weights attached to a given node. To characterize such heterogeneity, we use the disparity measure defined as

$$Y_i = \sum_j \left(\frac{w_{ij}}{s_i} \right)^2 , \tag{36}$$

where w_{ij} is the weight of the link between nodes i and j ($w_{ij} = 0$ if there is no link) and $s_i = \sum_j w_{ij}$ [10]. In Supplementary Figure 2**b**, we see that in the absence of coupling (i.e., $\alpha = 0.0$) the disparity scales as $Y_i \sim k_i^{-1}$ corresponding to the situation in which weights are roughly homogeneously distributed among the links [11]. On the other side of the spectrum, we see that under maximal coupling (i.e., $\alpha \lesssim 1$), the disparity decreases slower than k_i^{-1} meaning that weights are heterogeneous and that the large strength of nodes is due to a handful of links with large weights.

II. TEST OF THE TRIANGLE INEQUALITY

To test the triangle inequality in a given weighted complex network, we first find the parameters μ, β, and γ that best match the empirical topology. To achieve the optimal matching, we use the empiric sequence of degrees as input for the sequence of κ's so that the fluctuations in the tail of the degree distribution of the input network are preserved. The sequence is then used to generate different weighted networks as follows. From the empiric relation strength-degree, we measure the proportionality factor a and the exponent η, as well as the first and second moments of the strength distribution $\langle s \rangle$ and $\langle s^2 \rangle$. For fixed values of α and of the fluctuations of the pdf function $f(\cdot)$, $I_4 = \langle \epsilon^2 \rangle$, we generate a large number of synthetic weighted networks and measure the average value of $CV^2(s) = \mathrm{Var}[s^2]/\langle s \rangle^2$ and its ensemble fluctuations. From Sec. I A 3, we expect the average value of $CV^2(s)$ to scale linearly with $\langle \epsilon^2 \rangle$ as

$$CV^2(s) = \frac{\langle s^2 \rangle}{\langle s \rangle^2} - 1 = \frac{\Gamma(D/2) \langle \sigma^2/\kappa \rangle^2 I_5}{2\pi^{D/2} \delta \mu \langle \sigma \rangle^4 I_2^2 I_3^2} \langle \epsilon^2 \rangle . \tag{37}$$

The left hand side in this equation can be directly measured from the network. The right hand side depends linearly on the noise $\langle \epsilon^2 \rangle$ whereas its pre-factor depends both on the topology and on α_{real} through the integrals I_3 and I_5 (see Sec. I for technical details).

In Supplementary Figure 3**a**, we show $CV^2(s)$ as a function of $\langle \epsilon^2 \rangle$ and different values of α for one of the synthetic networks used in Supplementary Figure 4 with $\alpha_{\mathrm{origin}} = 0.4$ and noise

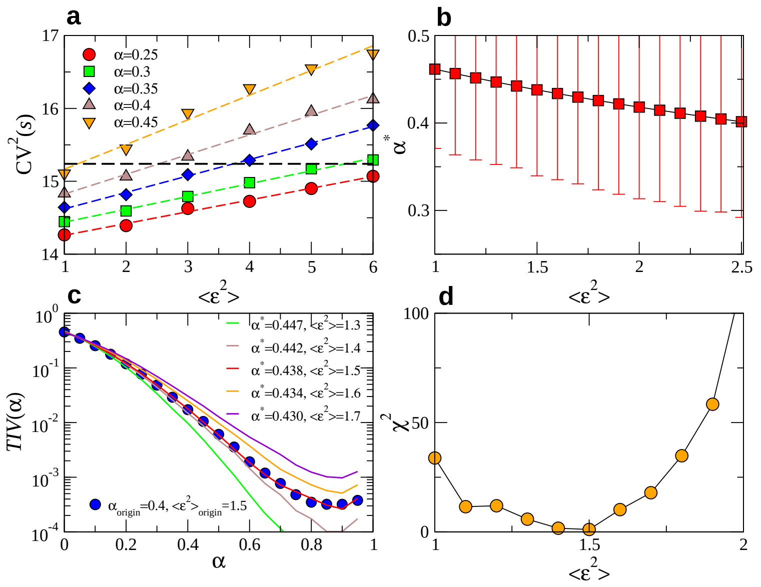

SUPPLEMENTARY FIGURE 3. **Illustration of the test of the triangle inequality** Test of the triangle inequality applied to a synthetic network generated with $\alpha_{\text{origin}} = 0.4$ and noise $\langle\epsilon^2\rangle_{\text{origin}} = 1.5$. **a**, the square of the coefficient of variation of nodes' strength as a function of the noise $\langle\epsilon^2\rangle$ in synthetic weighted networks with different values of α. The horizontal dashed line is the empirical value measured in the input network. **b**, values of α^* as a function of the noise obtained from the intersection of the dashed line in **a** with the synthetic curves. **c**, $TIV(\alpha)$ curves for synthetic networks with the values of α^* and $\langle\epsilon^2\rangle$ from **b** compared to the same function for the input network. **d**, χ^2 statistics obtained from the comparison of function $TIV(\alpha)$ between the model and input network.

$\langle\epsilon^2\rangle_{\text{origin}} = 1.5$. The intersection of these curves with the empirical value of $CV^2(s)$ defines a collection of α's as a function of the noise, $\alpha^*\left(\langle\epsilon^2\rangle\right)$, which become the potential candidates to be the estimate of α_{real} (see Supplementary Figure 3**b**). Finally, for each pair $(\langle\epsilon^2\rangle, \alpha^*)$ in Supplementary Figure 3**b** we measure the function $TIV(\alpha)$ and compare it with the same function measured in the input network (see Supplementary Figure 3**c**). The comparison is performed by measuring the standard χ^2 statistics. The inferred value of α_{real} corresponds to the value of $\alpha^*(\langle\epsilon^2\rangle)$ minimizing the value of χ^2 (see Supplementary Figure 3**d**). To find a lower bound of the inferred value of α_{real}, we use the ensemble fluctuations of $CV^2(s)$. For any fixed value of $\langle\epsilon^2\rangle$, the lower bound of α^* is the value of α that is still able to reproduce the empirical value of $CV^2(s)$ (see Supplementary Figure 3**b**). The lower bound for the inferred value of α_{real} is the lower bound of α^* that corresponds to the optimal value of the noise $\langle\epsilon^2\rangle$ in Supplementary Figure 3**d**. Supplementary Figure 4 shows the result of this method in the case of synthetic networks with different values of α and noise levels. As it can be seen, the inferred values of α match the true values in most of the cases. In fact, these fluctuations are due to the fact that the noise measured in the original synthetic network does not necessarily equal the typical noise of the ensemble of networks generated using the same parameters. Our method also allows us to find lower bounds on the inferred values, as shown by the grey areas in Supplementary Figure 4. The most remarkable aspect of the test is that it can be performed without any explicit embedding of the network and, thus, it can be readily applied to real networks for which an embedding is not available.

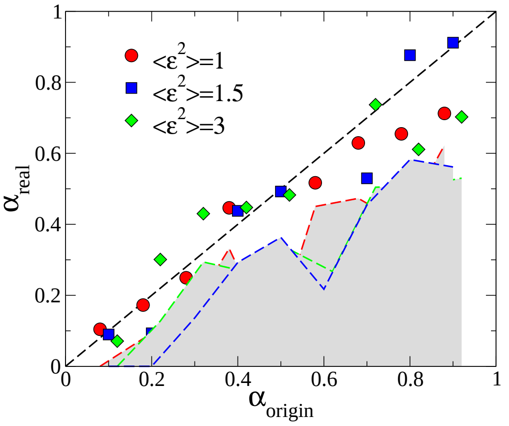

SUPPLEMENTARY FIGURE 4. **Validation of the test of the triangle inequality** α_{real} vs. α_{origin} for synthetic networks generated with the model for different values of noise $\langle \epsilon^2 \rangle$. In all cases, network topologies are generated with $\gamma = 2.5$, $\beta = 2$, $\eta = 1.2$, $\langle k \rangle = 10$, and $N = 10^4$. The solid grey area indicates the lower bounds found by the method.

A. Violation of the triangle inequality

We expect the violation of the triangle inequality to depend essentially on the level of noise in the system $\langle \epsilon^2 \rangle$ through the term in the right hand side of Eq. (7) in the main text. To a lesser extent, the violation may also be due to the fact that the hidden variables κ and σ are approximated by the actual degree and the strength, respectively, of nodes. For most of the analysed real networks, the percentage of violations is very small (of the order of few percent) whereas in the case of the cargo ships network it is close to 20%, due to the high level of noise present in the system. In short, our model predicts that there should not be any dependence on the degree in the nodes belonging to triangles that violate the triangle inequality. To test this prediction, we have measured explicitly the average degree of such nodes as compared to the average degree of nodes in all triangles (see Supplementary Figure 5). In many cases the average degree is very similar, thus confirming our prediction. The largest discrepancy is found in the metabolic network. However, notice that this network has a very small percentage of violations, which makes it more prone to statistical fluctuations.

B. Behaviour of $TIV(\alpha)$ with $\alpha \sim 1$

The increase of $TIV(\alpha)$ close to $\alpha = 1$ on Supplementary Figure 3 and on Figs. **3a**–**b** in the main text is expected and is in fact an artefact of Eq. (5) and of our our choice of the probability of connection [i.e., Eq. (30)]. Indeed, substituting Eq. (23) in Eq. (7) in the main text and neglecting the noise term (whose mean value is close to zero) we obtain

$$\ln\left[\frac{\omega_{ij}\omega_{jk}}{\omega_{ik}}\left(\frac{\kappa_j}{\sigma_j}\right)^2\right] \leq \frac{R}{2}\alpha + \ln\left(\sin\left[\frac{(1-\alpha)\pi}{\beta}\right]\right) + \ln\left(\frac{\beta}{2\pi\mu\langle\sigma\rangle}\right) . \tag{38}$$

Supplementary Figure 6 shows the behaviour of α-dependent terms of the right hand side of Eq. (38) for the real networks considered in the main text. For the low values of α, we see that the right hand side of Eq. (38) is an increasing function which implies that $TIV(\alpha)$ decreases with increasing α (i.e., it is *more and more difficult* to violate the triangle inequality as α increases). However, all curves reach a plateau at $\alpha \simeq 0.8$ after which they start to decrease. As expected, these plateaus

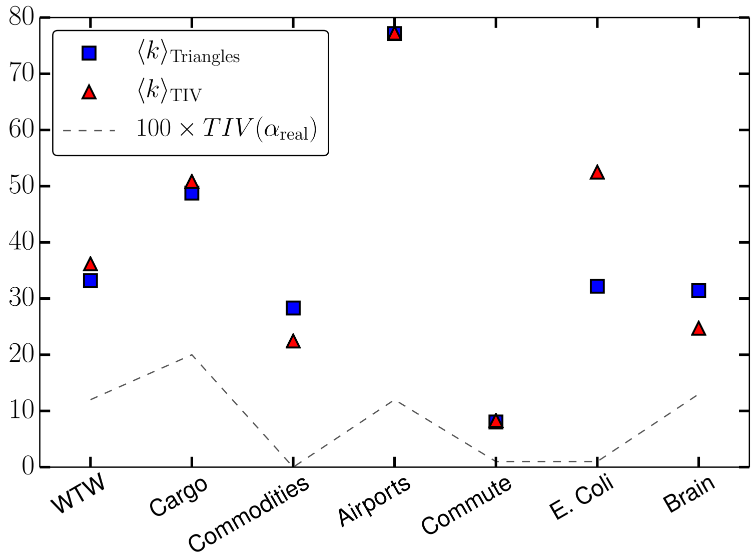

SUPPLEMENTARY FIGURE 5. **Further properties of triangles** Average degree of nodes in triangles that violate the triangle inequality ($\langle k \rangle_{\text{TIV}}$) and in all triangles ($\langle k \rangle_{\text{Triangle}}$) for the networks considered in the main text. The average is performed by sampling over triangles which implies that the degree of a node is weighted by the number of triangles to which it participates (as in Fig. 1 of the main text). The dashed line shows the fraction of triangles that violate the triangle inequality when using the inferred value α_{real}.

correspond to the points where the $TIV(\alpha)$ start to increase (for some networks this increase is not visible due to the linear scale of the y axis).

C. Application to real networks

We applied this methodology to the real networks mentioned in the paper and Supplementary Table 1 shows the parameters thus inferred (see also Fig. 3 in the main text). The comparison between the properties of networks generated using these parameters with the ones of the original real networks is shown on Supplementary Figures 7–12. Besides some expected fluctuations inherent to the model (i.e., only one synthetic network is used for each figure), these figures confirm that the model can reproduce many topological and weighted features observed in real complex networks.

III. COMPARISON WITH OTHER MODELS

We present further evidence to support the claim that our model is the most accurate approach to model real weighted complex networks. To do so, we show the results obtained by using the two models introduced in Refs. [6] and [12], as well as a new one that generalizes them. These models use the original network topology randomized under the constraints of preserving the degree sequence and the average clustering coefficient using the software developed in Refs. [13, 14]. Weights are then assigned to each link according to the following rules:

- **model A:** $w_{ij} \propto (k_i k_j)^{\theta}$, where k_i and k_j are the degrees of nodes i and j, respectively;

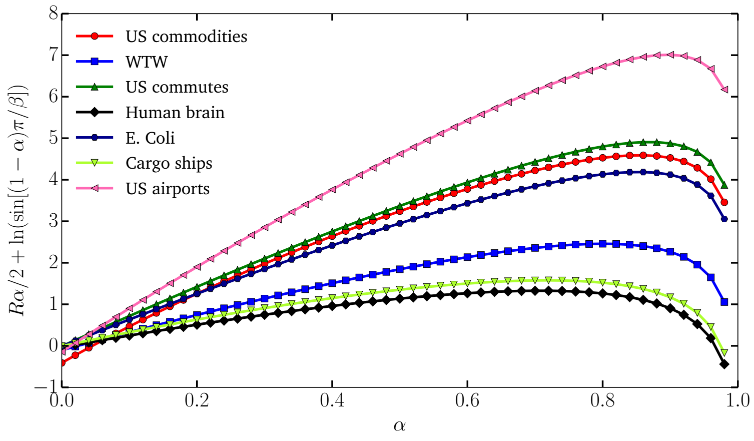

SUPPLEMENTARY FIGURE 6. **Behaviour of the violation threshold** α-dependent terms of the right hand side of Eq. (38) as a function of α for the real networks considered in the main text.

- **model B:** $w_{ij} \propto (c_i c_j)^{\delta}$, where c_i and c_j are the clustering coefficient of nodes i and j, respectively;
- **model C:** $w_{ij} \propto (k_i k_j)^{\mu} (c_i c_j)^{\nu}$. This model accounts for the fact that weights among high degree nodes are higher but also that weights among highly clustered nodes are also higher.

The exponents θ, δ, μ and ν are chosen as those minimizing the χ^2 statistic for the corresponding dataset (see the captions of Supplementary Figures 14–41 for the inferred values).

Although the three models reproduce exactly the degree sequence, the degree-dependent clustering of the randomized networks is never better than the one obtained with our model. We find that models A and C can reproduce fairly well the strength distributions, or at least their general shapes. This is due to the strong influence of the topology over the weighted organization, and it illustrates well the reason why we factorized the weights on Fig. 1 in the main text to account for the effect of the topology. However, except for the world trade web and US airports network, we find that the three models reproduce poorly the weight distributions and the disparities. This is not particularly surprising in the case of the US airports network since our model predicts a weaker dependence on the metric space, leaving weights mainly as a function of the degree of nodes. Similarly, it is not surprising in the case of the world trade web given its small size. Nevertheless, even though some of the local properties can be reproduced in some of the networks by the three models, Supplementary Figures 14–41 show that none of them can reproduce the $TIV(\alpha)$ curves observed for the real networks, suggesting that our assumption about the metric origin of weights is a much better explanation of the real data.

**Name**	$\rho_{m,\omega}$	$\rho_{m,\omega^{\text{norm}}}$	β	α	$\langle\epsilon^2\rangle$	η	a	**Suppl. Figure**
World Trade	0.68	0.10	2.5	0.41	1.3	1.63	3772	7
Cargo ships	0.19	0.10	1.85	0.65	1.7	1.05	83	8
US Commodities	0.24	0.05	1.3	0.65	1.2	1.22	3045	9
US Airports	0.72	0.03	1.4	0.15	1.4	1.72	10000	10
US Commute	0.51	0.17	2.2	0.59	1.4	2.02	719	11
E. Coli	0.73	0.45	2.2	0.45	1.3	1.09	1	12
Human brain	0.27	0.23	2.8	0.45	1.3	0.86	0.015	13

SUPPLEMENTARY TABLE 1. **Parameters and information about the datasets** Pearson correlation coefficients between the multiplicity, m, and the weight and normalized weight of links in the real networks considered in the paper. Also, the parameters used to reproduce real networks with our model (see the main text for description).

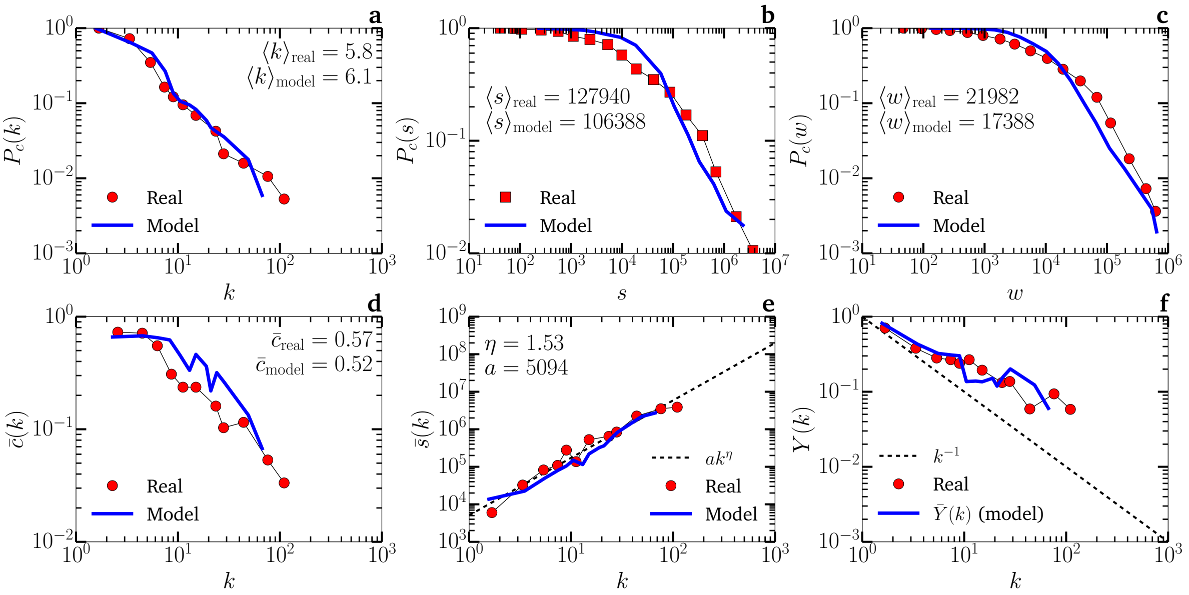

SUPPLEMENTARY FIGURE 7. **Predictions by the model introduced in Sec. I.** Comparison between topological and weighted properties of the WTW (symbols) and a synthetic network generated by the model with the parameters given in Supplementary Table 1 (solid lines). **a**, complementary cumulative degree distribution. **b**, complementary cumulative strength distribution. **c**, complementary cumulative weight distribution of links. **d**, degree-dependent clustering coefficient. **e**, average strength of nodes of degree k. **f**, disparity of nodes as a function of their degree.

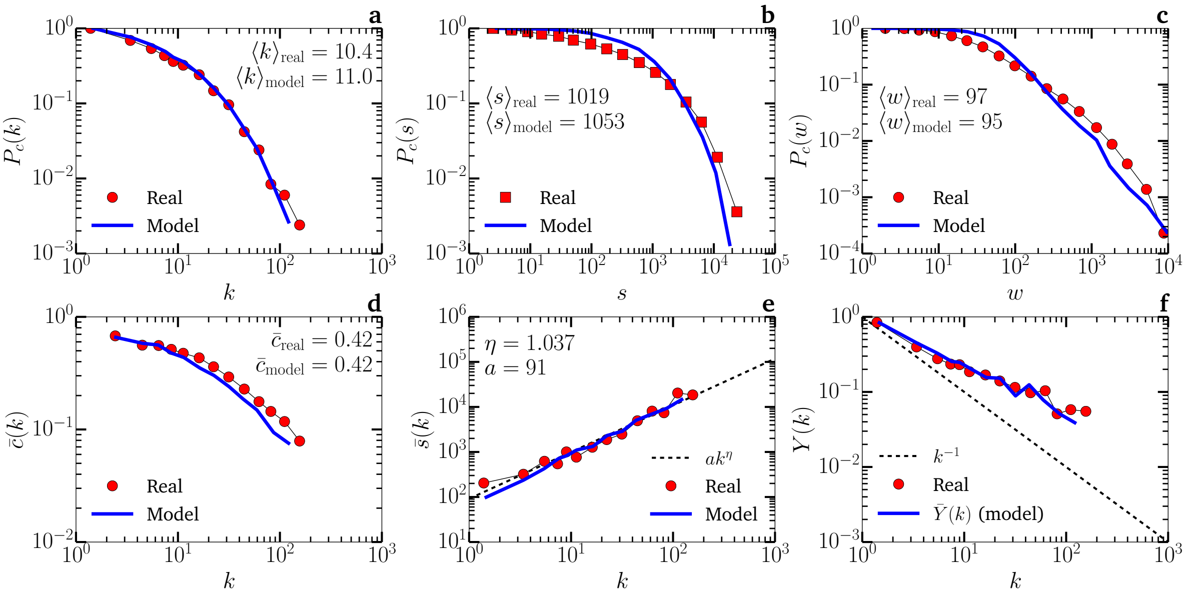

SUPPLEMENTARY FIGURE 8. **Predictions by the model introduced in Sec. I.** Comparison between topological and weighted properties of the Cargo ships network (symbols) and a synthetic network generated by the model with the parameters given in Supplementary Table 1 (solid lines). **a**, complementary cumulative degree distribution. **b**, complementary cumulative strength distribution. **c**, complementary cumulative weight distribution of links. **d**, degree-dependent clustering coefficient. **e**, average strength of nodes of degree k. **f**, disparity of nodes as a function of their degree.

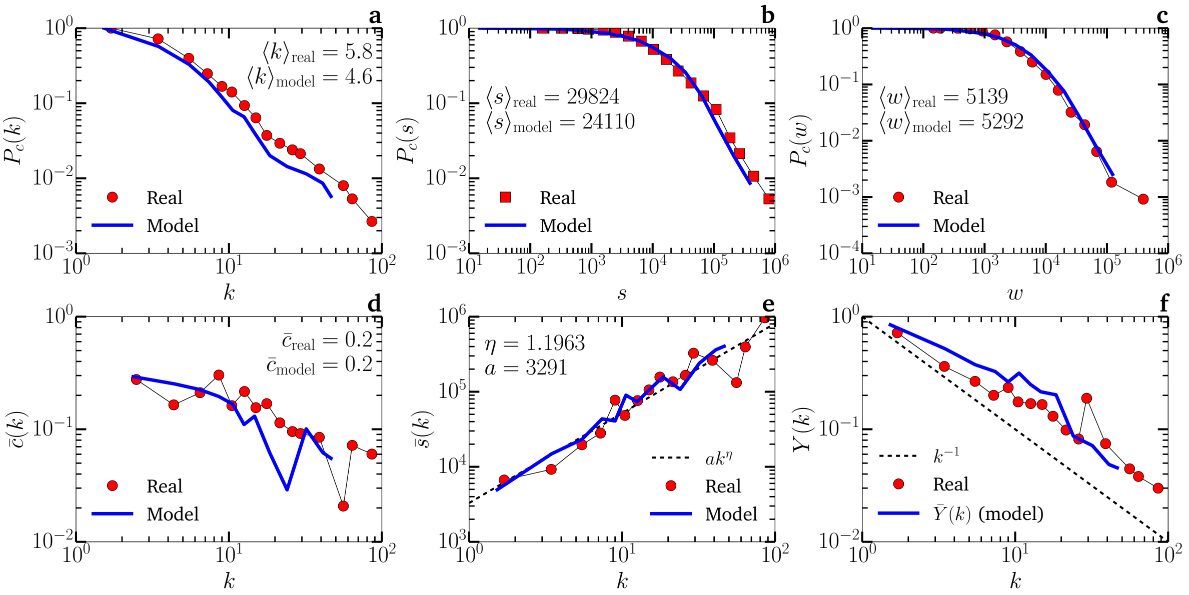

SUPPLEMENTARY FIGURE 9. **Predictions by the model introduced in Sec. I.** Comparison between topological and weighted properties of the US Commodities network (symbols) and a synthetic network generated by the model with the parameters given in Supplementary Table 1 (solid lines). **a**, complementary cumulative degree distribution. **b**, complementary cumulative strength distribution. **c**, complementary cumulative weight distribution of links. **d**, degree-dependent clustering coefficient. **e**, average strength of nodes of degree k. **f**, disparity of nodes as a function of their degree.

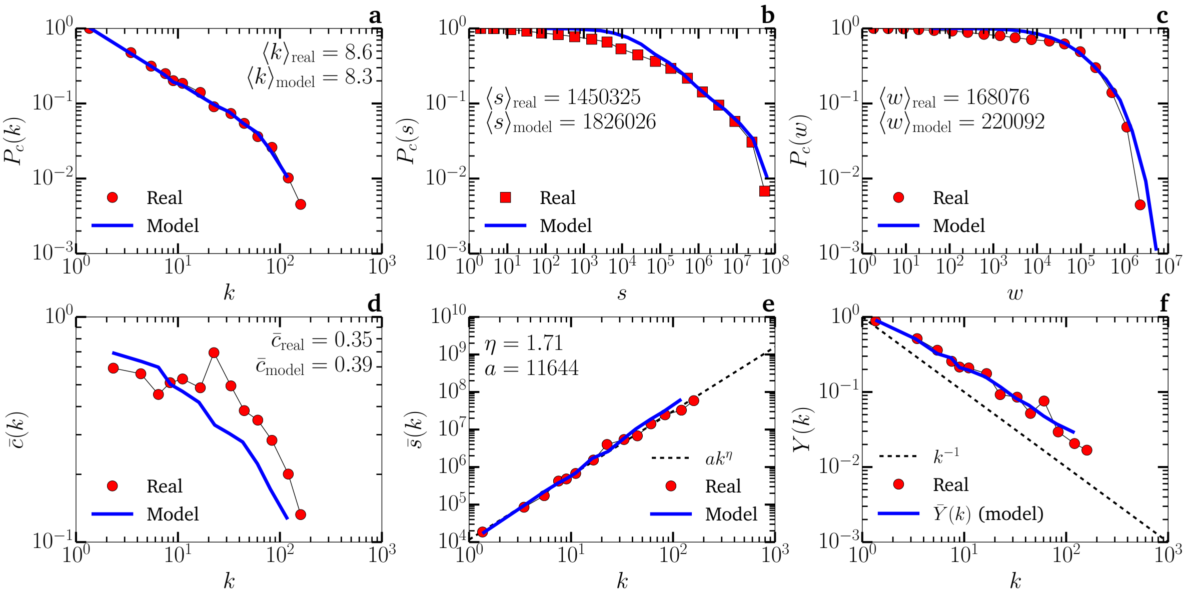

SUPPLEMENTARY FIGURE 10. **Predictions by the model introduced in Sec. I.** Comparison between topological and weighted properties of the US airports network (symbols) and a synthetic network generated by the model with the parameters given in Supplementary Table 1 (solid lines). **a**, complementary cumulative degree distribution. **b**, complementary cumulative strength distribution. **c**, complementary cumulative weight distribution of links. **d**, degree-dependent clustering coefficient. **e**, average strength of nodes of degree k. **f**, disparity of nodes as a function of their degree.

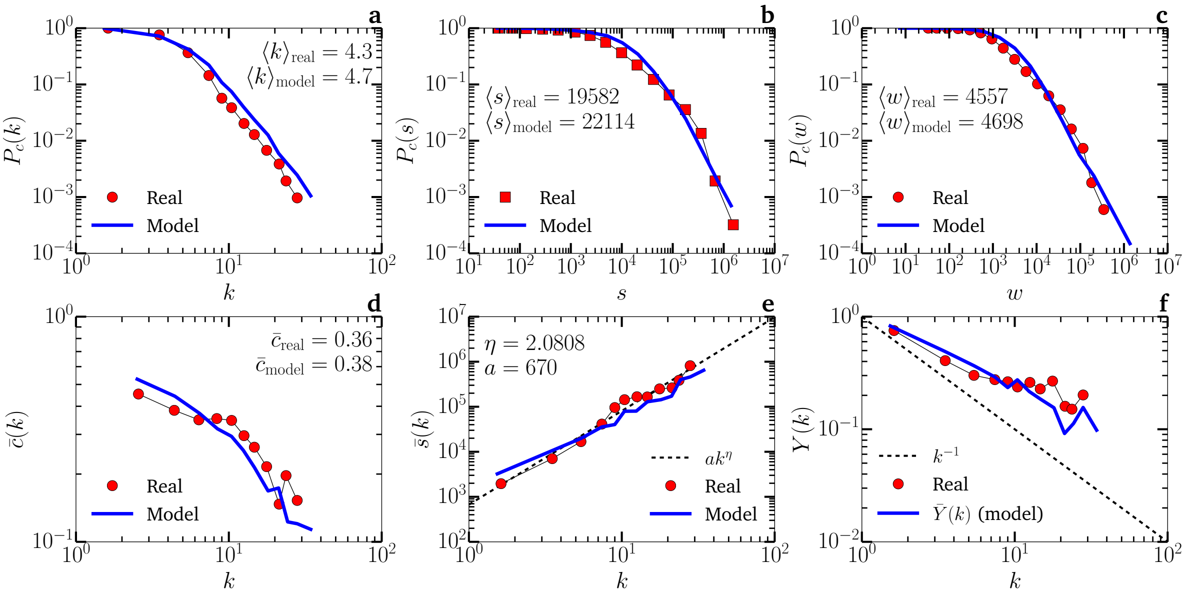

SUPPLEMENTARY FIGURE 11. **Predictions by the model introduced in Sec. I.** Comparison between topological and weighted properties of the US Commute network (symbols) and a synthetic network generated by the model with the parameters given in Supplementary Table 1 (solid lines). **a**, complementary cumulative degree distribution. **b**, complementary cumulative strength distribution. **c**, complementary cumulative weight distribution of links. **d**, degree-dependent clustering coefficient. **e**, average strength of nodes of degree k. **f**, disparity of nodes as a function of their degree.

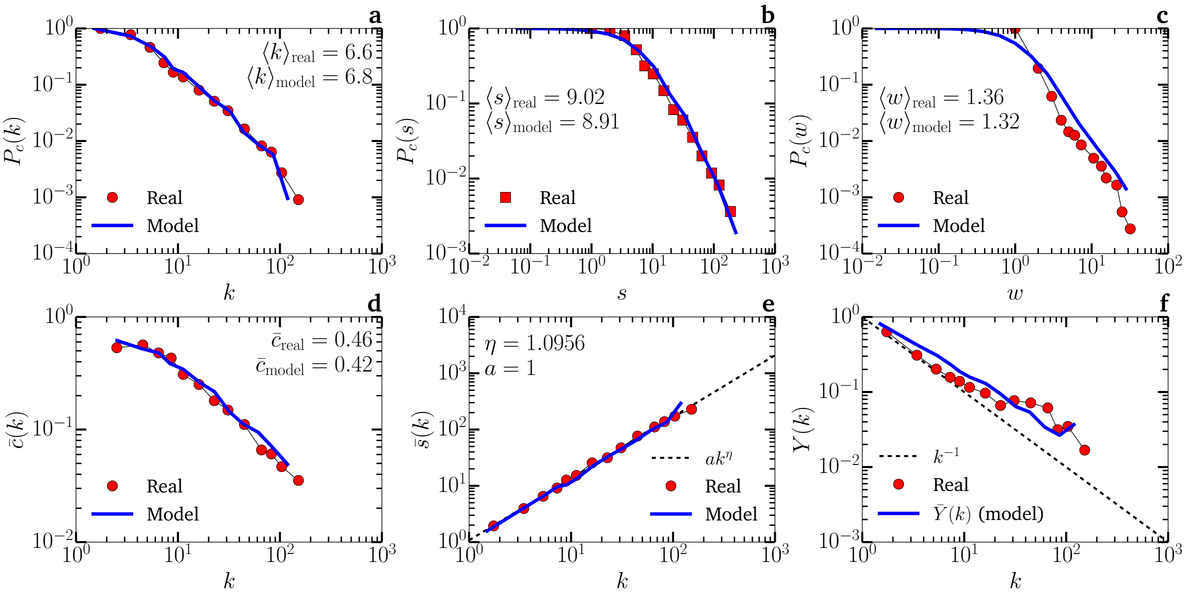

SUPPLEMENTARY FIGURE 12. **Predictions by the model introduced in Sec. I.** Comparison between topological and weighted properties of the iJO1366 *E. Coli* metabolic network (symbols) and a synthetic network generated by the model with the parameters given in Supplementary Table 1 (solid lines). **a**, complementary cumulative degree distribution. **b**, complementary cumulative strength distribution. **c**, complementary cumulative weight distribution of links. **d**, degree-dependent clustering coefficient. **e**, average strength of nodes of degree k. **f**, disparity of nodes as a function of their degree.

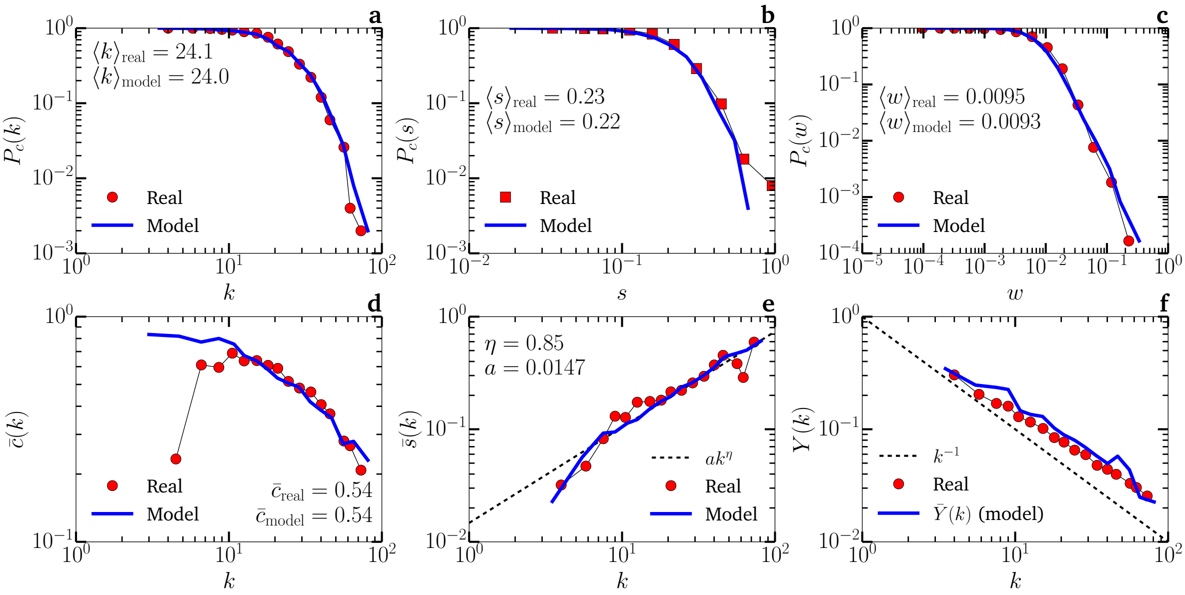

SUPPLEMENTARY FIGURE 13. **Predictions by the model introduced in Sec. I.** Comparison between topological and weighted properties of the Human brain network (symbols) and a synthetic network generated by the model with the parameters given in Supplementary Table 1 (solid lines). **a**, complementary cumulative degree distribution. **b**, complementary cumulative strength distribution. **c**, complementary cumulative weight distribution of links. **d**, degree-dependent clustering coefficient. **e**, average strength of nodes of degree k. **f**, disparity of nodes as a function of their degree.

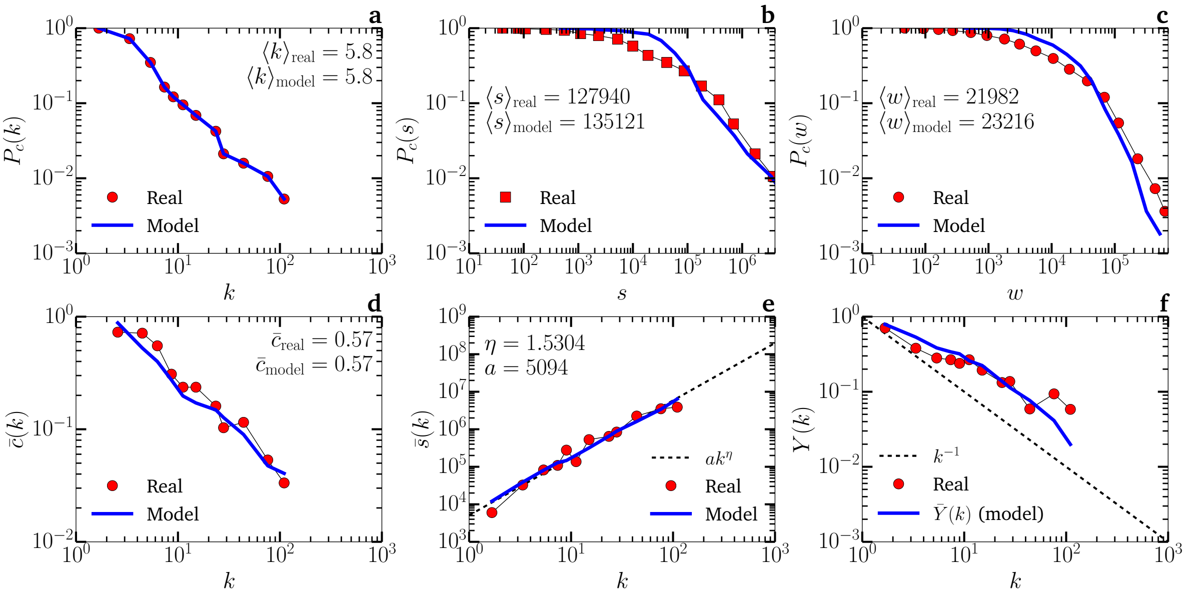

SUPPLEMENTARY FIGURE 14. **Predictions by Model A.** Comparison between topological and weighted properties of the WTW (symbols) and a synthetic network generated by model A with $\theta = 0.9$ (solid lines). **a**, complementary cumulative degree distribution. **b**, complementary cumulative strength distribution. **c**, complementary cumulative weight distribution of links. **d**, degree-dependent clustering coefficient. **e**, average strength of nodes of degree k. **f**, disparity of nodes as a function of their degree.

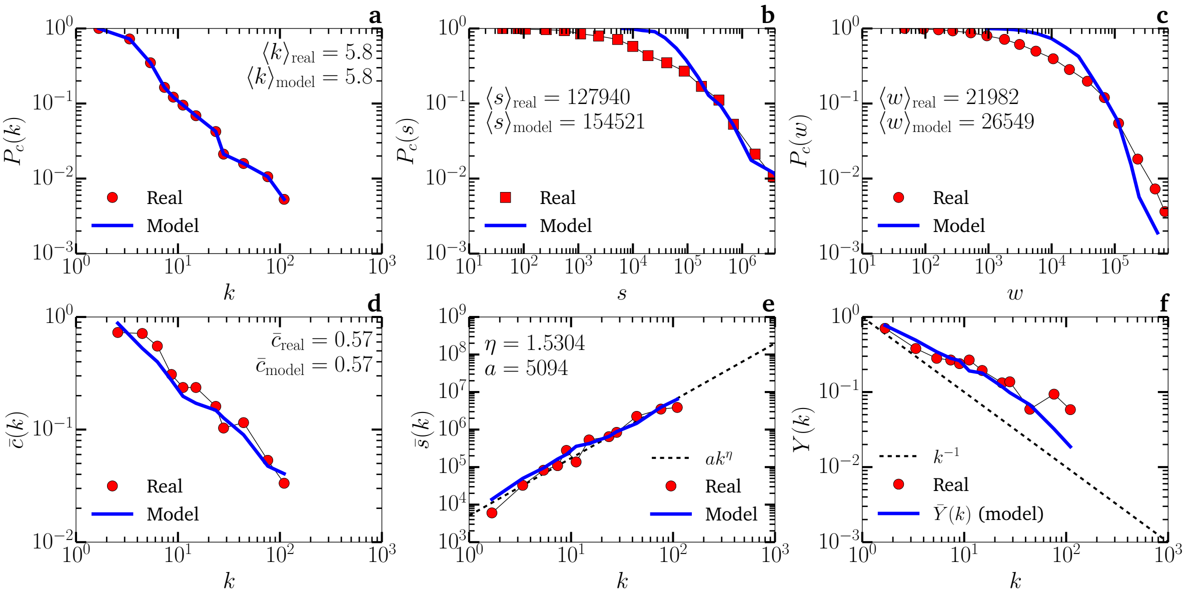

SUPPLEMENTARY FIGURE 15. **Predictions by Model B.** Comparison between topological and weighted properties of the WTW (symbols) and a synthetic network generated by model B with $\delta = -1.01$ (solid lines). **a**, complementary cumulative degree distribution. **b**, complementary cumulative strength distribution. **c**, complementary cumulative weight distribution of links. **d**, degree-dependent clustering coefficient. **e**, average strength of nodes of degree k. **f**, disparity of nodes as a function of their degree.

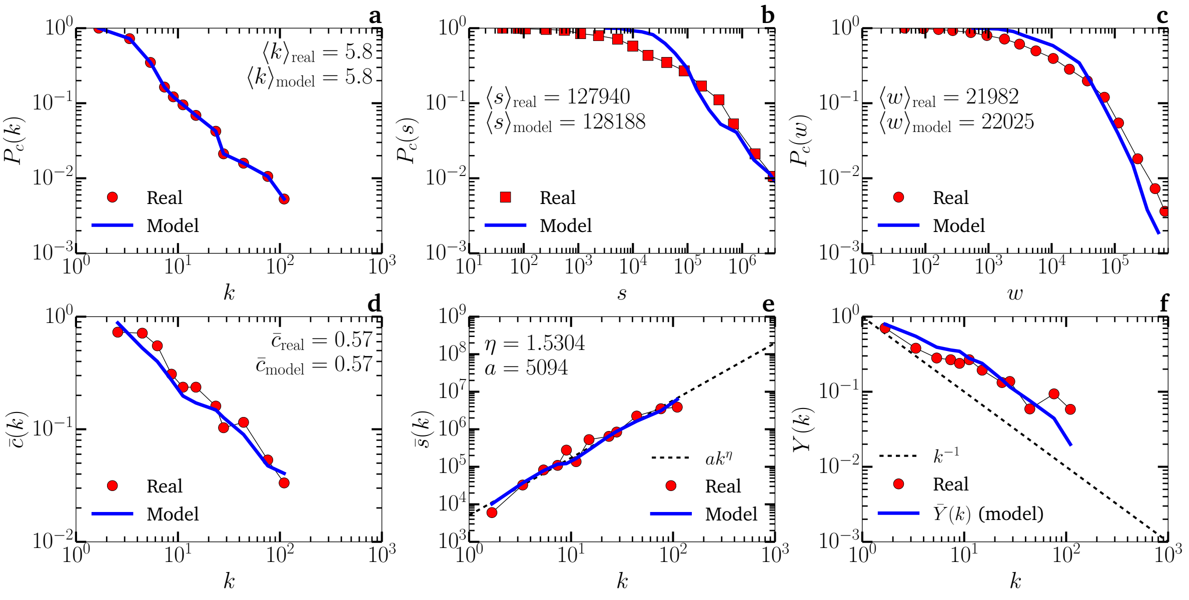

SUPPLEMENTARY FIGURE 16. **Predictions by Model C.** Comparison between topological and weighted properties of the WTW (symbols) and a synthetic network generated by model C with $\mu = 1.3$ and $\nu = 0.475$ (solid lines). **a**, complementary cumulative degree distribution. **b**, complementary cumulative strength distribution. **c**, complementary cumulative weight distribution of links. **d**, degree-dependent clustering coefficient. **e**, average strength of nodes of degree k. **f**, disparity of nodes as a function of their degree.

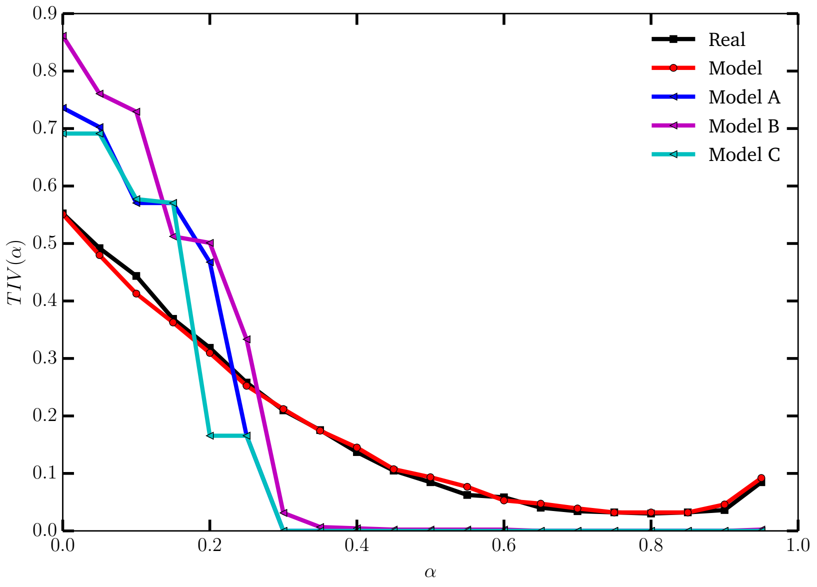

SUPPLEMENTARY FIGURE 17. **Triangle inequality violation spectrum.** Comparison between $TIV(\alpha)$ curves measured for the real WTW, our model and the models A, B and C with the exponents given in the caption of Supplementary Figures 14–16.

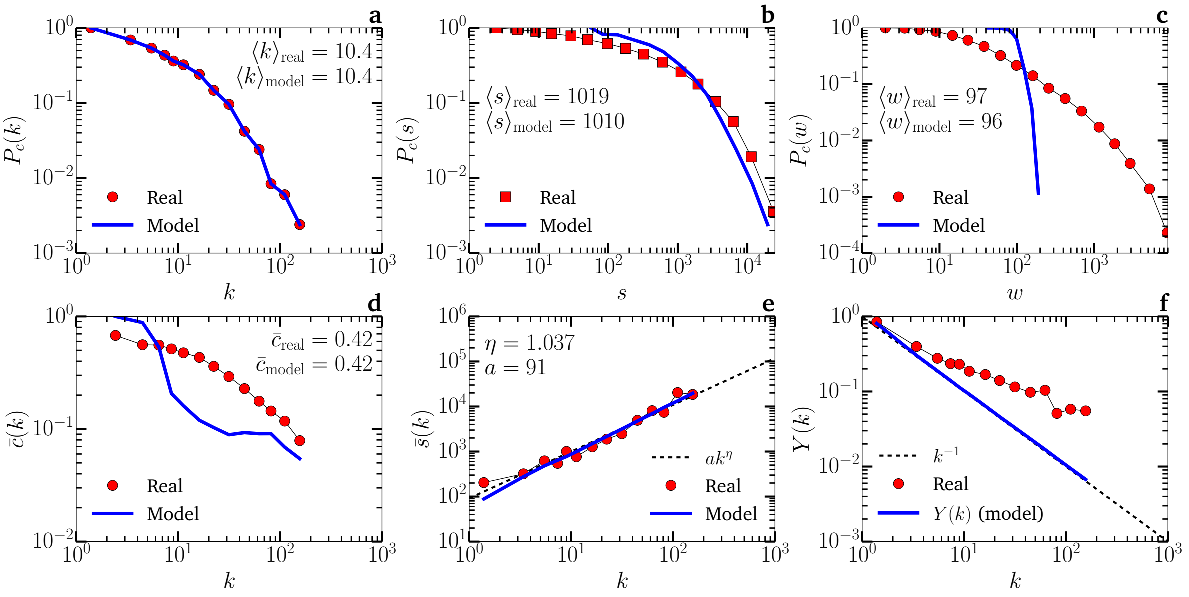

SUPPLEMENTARY FIGURE 18. **Predictions by Model A.** Comparison between topological and weighted properties of the Cargo ships network (symbols) and a synthetic network generated by model A with $\theta = 0.18$ (solid lines). **a**, complementary cumulative degree distribution. **b**, complementary cumulative strength distribution. **c**, complementary cumulative weight distribution of links. **d**, degree-dependent clustering coefficient. **e**, average strength of nodes of degree k. **f**, disparity of nodes as a function of their degree.

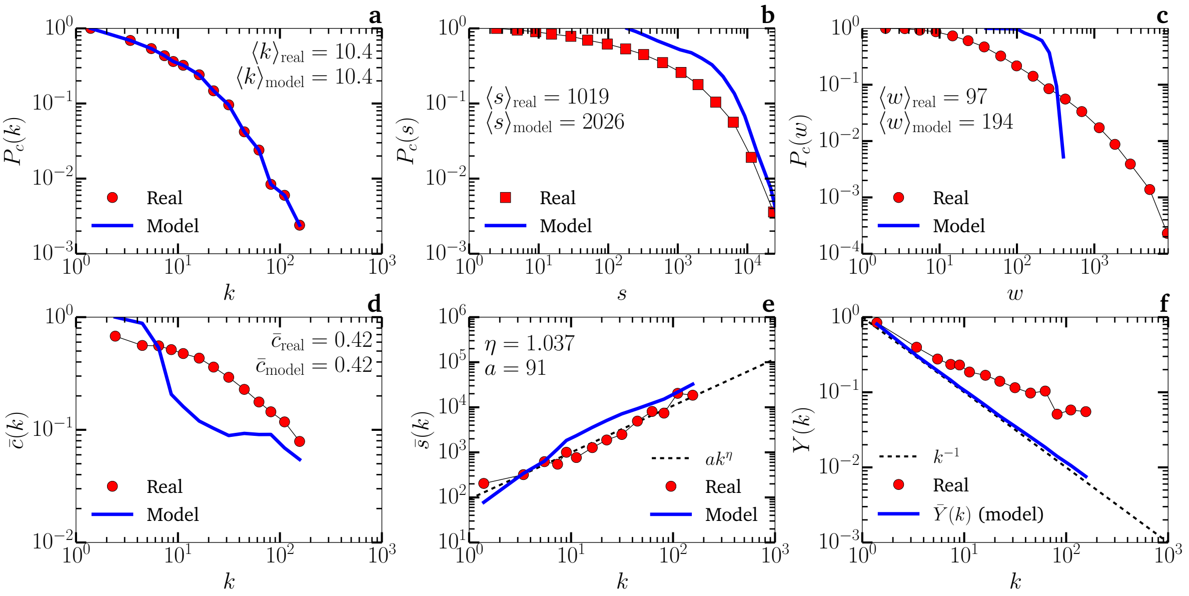

SUPPLEMENTARY FIGURE 19. **Predictions by Model B.** Comparison between topological and weighted properties of the Cargo ships network (symbols) and a synthetic network generated by model B with $\delta = -0.39$ (solid lines). **a**, complementary cumulative degree distribution. **b**, complementary cumulative strength distribution. **c**, complementary cumulative weight distribution of links. **d**, degree-dependent clustering coefficient. **e**, average strength of nodes of degree k. **f**, disparity of nodes as a function of their degree.

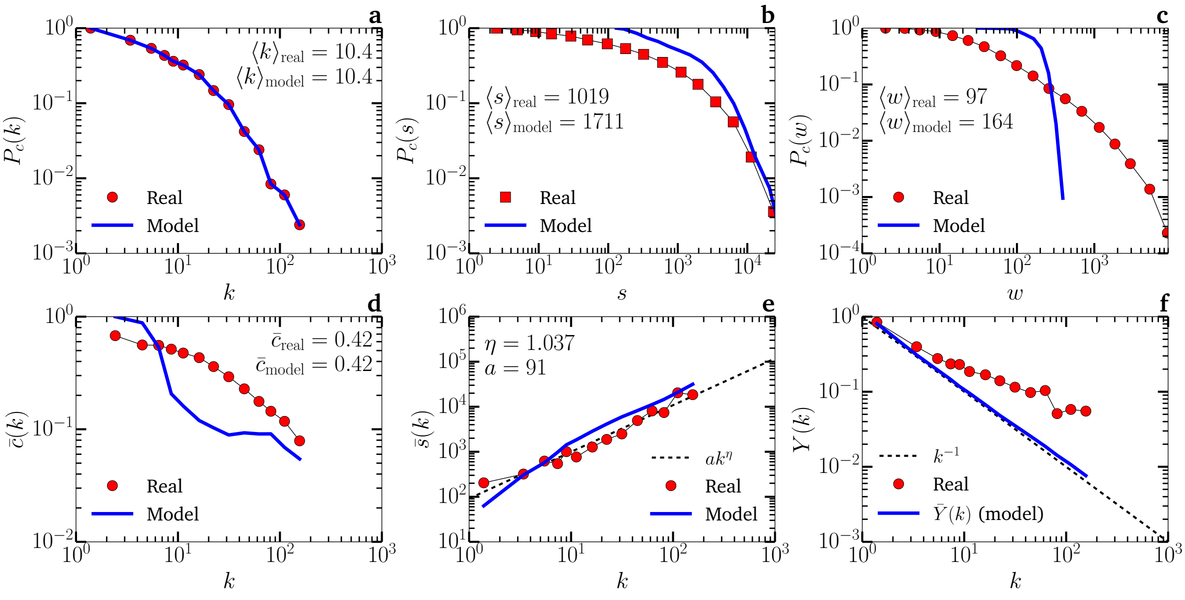

SUPPLEMENTARY FIGURE 20. **Predictions by Model C.** Comparison between topological and weighted properties of the Cargo ships network (symbols) and a synthetic network generated by model C with $\mu = 1.0$ and $\nu = -0.3$ (solid lines). **a**, complementary cumulative degree distribution. **b**, complementary cumulative strength distribution. **c**, complementary cumulative weight distribution of links. **d**, degree-dependent clustering coefficient. **e**, average strength of nodes of degree k. **f**, disparity of nodes as a function of their degree.

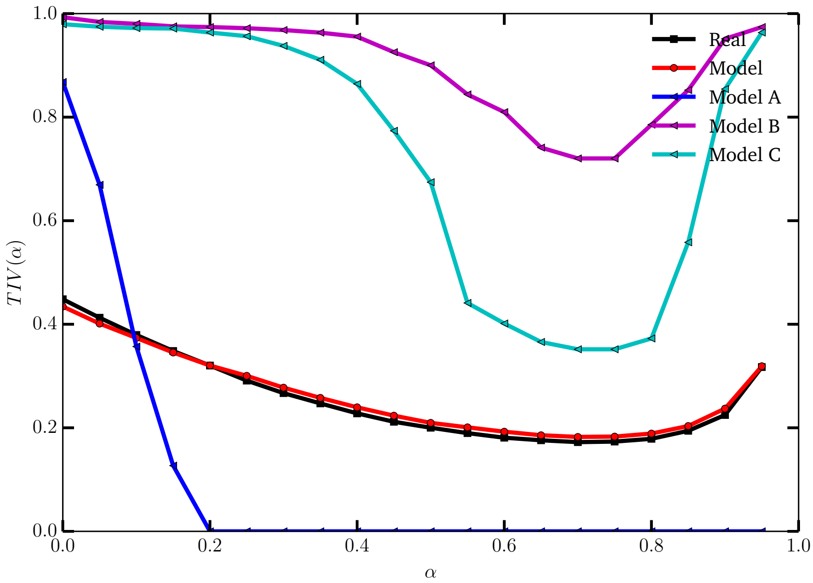

SUPPLEMENTARY FIGURE 21. **Triangle inequality violation spectrum.** Comparison between $TIV(\alpha)$ curves measured for the real Cargo ships network, our model and the models A, B and C with the exponents given in the caption of Supplementary Figures 18–20.

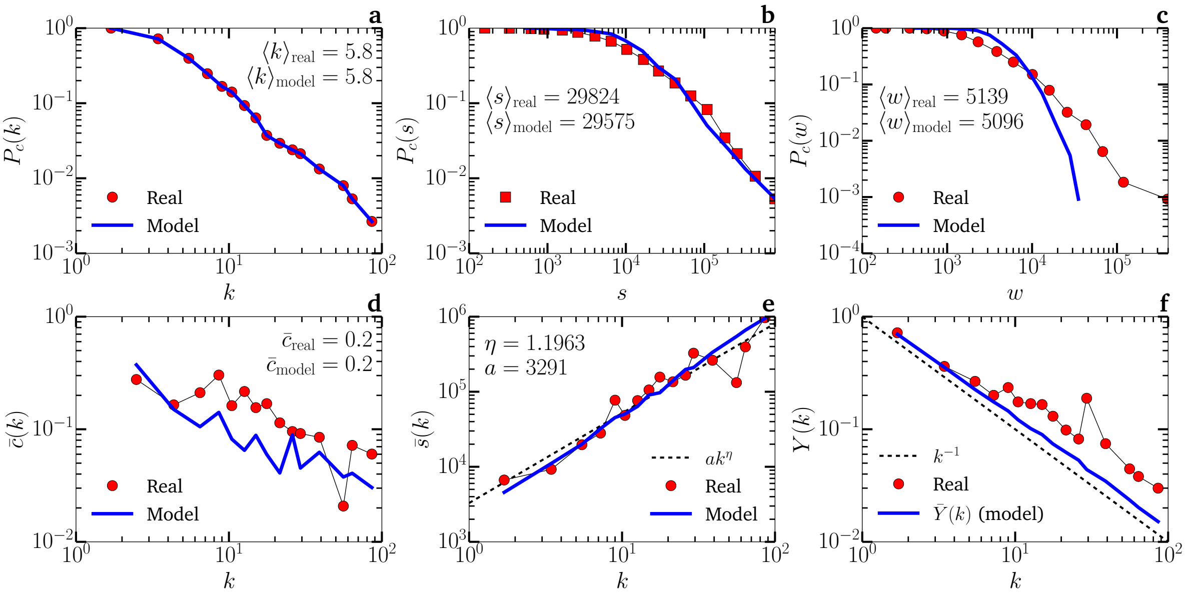

SUPPLEMENTARY FIGURE 22. **Predictions by Model A.** Comparison between topological and weighted properties of the US Commodities network (symbols) and a synthetic network generated by model A with $\theta = 0.51$ (solid lines). **a**, complementary cumulative degree distribution. **b**, complementary cumulative strength distribution. **c**, complementary cumulative weight distribution of links. **d**, degree-dependent clustering coefficient. **e**, average strength of nodes of degree k. **f**, disparity of nodes as a function of their degree.

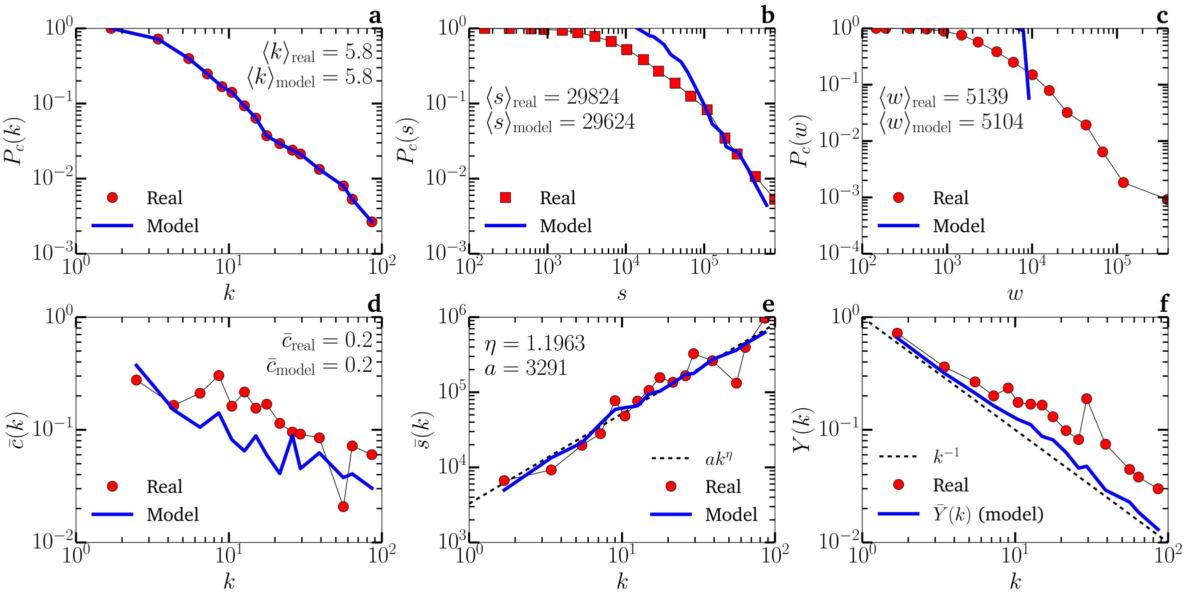

SUPPLEMENTARY FIGURE 23. **Predictions by Model B.** Comparison between topological and weighted properties of the US Commodities network (symbols) and a synthetic network generated by model B with $\delta = -0.07$ (solid lines). **a**, complementary cumulative degree distribution. **b**, complementary cumulative strength distribution. **c**, complementary cumulative weight distribution of links. **d**, degree-dependent clustering coefficient. **e**, average strength of nodes of degree k. **f**, disparity of nodes as a function of their degree.

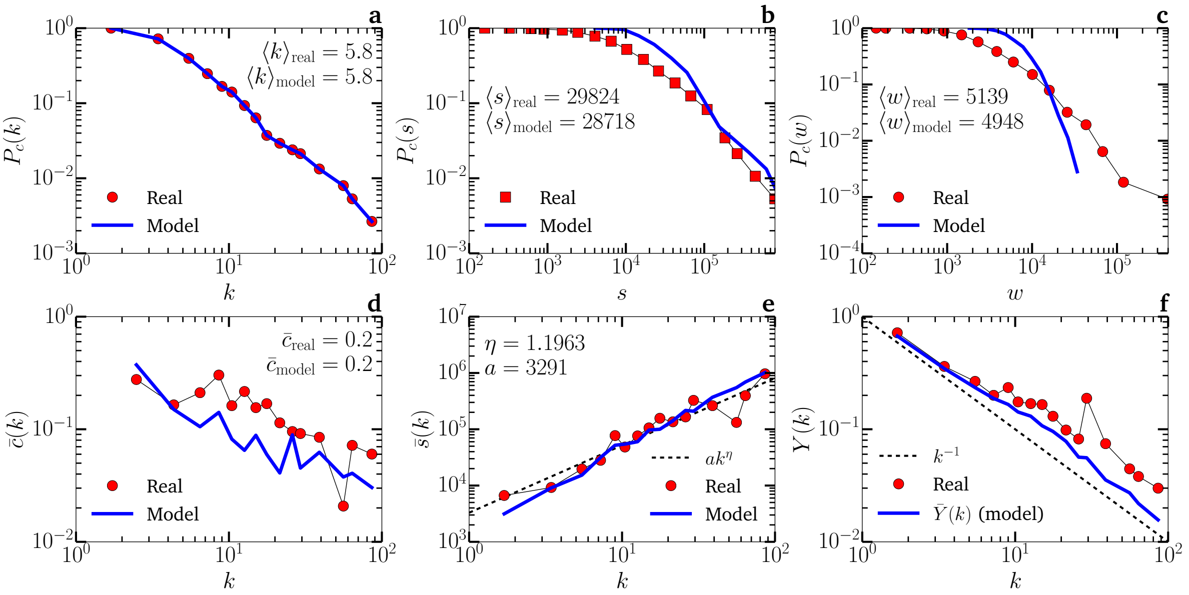

SUPPLEMENTARY FIGURE 24. **Predictions by Model C.** Comparison between topological and weighted properties of the US Commodities network (symbols) and a synthetic network generated by model C with $\mu = 0.425$ and $\nu = -0.025$ (solid lines). **a**, complementary cumulative degree distribution. **b**, complementary cumulative strength distribution. **c**, complementary cumulative weight distribution of links. **d**, degree-dependent clustering coefficient. **e**, average strength of nodes of degree k. **f**, disparity of nodes as a function of their degree.

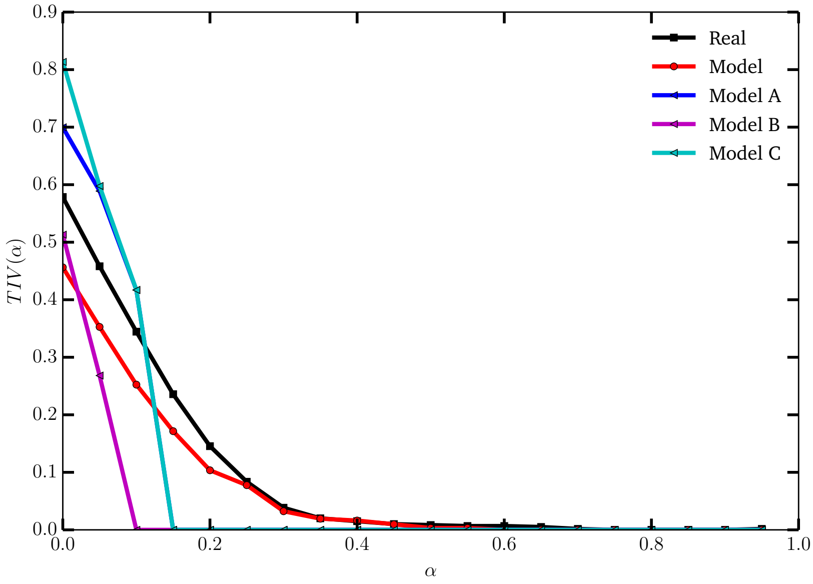

SUPPLEMENTARY FIGURE 25. **Triangle inequality violation spectrum.** Comparison between $TIV(\alpha)$ curves measured for the real US Commodities network, our model and the models A, B and C with the exponents given in the caption of Supplementary Figures 22–24.

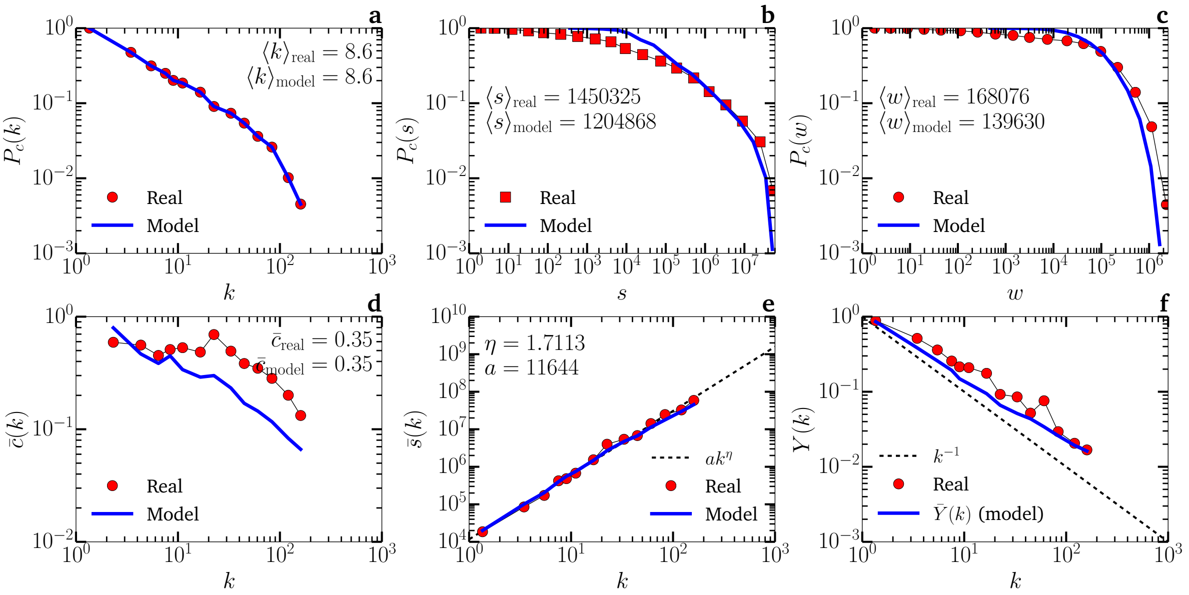

SUPPLEMENTARY FIGURE 26. **Predictions by Model A.** Comparison between topological and weighted properties of the US airports network (symbols) and a synthetic network generated by model A with $\theta = 0.89$ (solid lines). **a**, complementary cumulative degree distribution. **b**, complementary cumulative strength distribution. **c**, complementary cumulative weight distribution of links. **d**, degree-dependent clustering coefficient. **e**, average strength of nodes of degree k. **f**, disparity of nodes as a function of their degree.

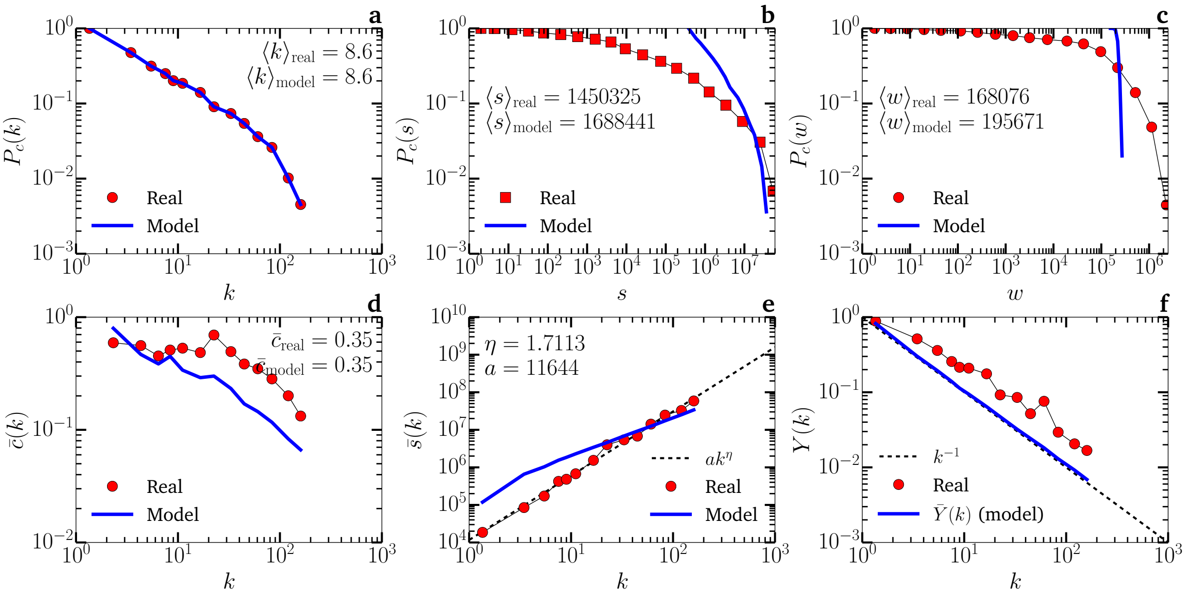

SUPPLEMENTARY FIGURE 27. **Predictions by Model B.** Comparison between topological and weighted properties of the US airports network (symbols) and a synthetic network generated by model B with $\delta = -0.12$ (solid lines). **a**, complementary cumulative degree distribution. **b**, complementary cumulative strength distribution. **c**, complementary cumulative weight distribution of links. **d**, degree-dependent clustering coefficient. **e**, average strength of nodes of degree k. **f**, disparity of nodes as a function of their degree.

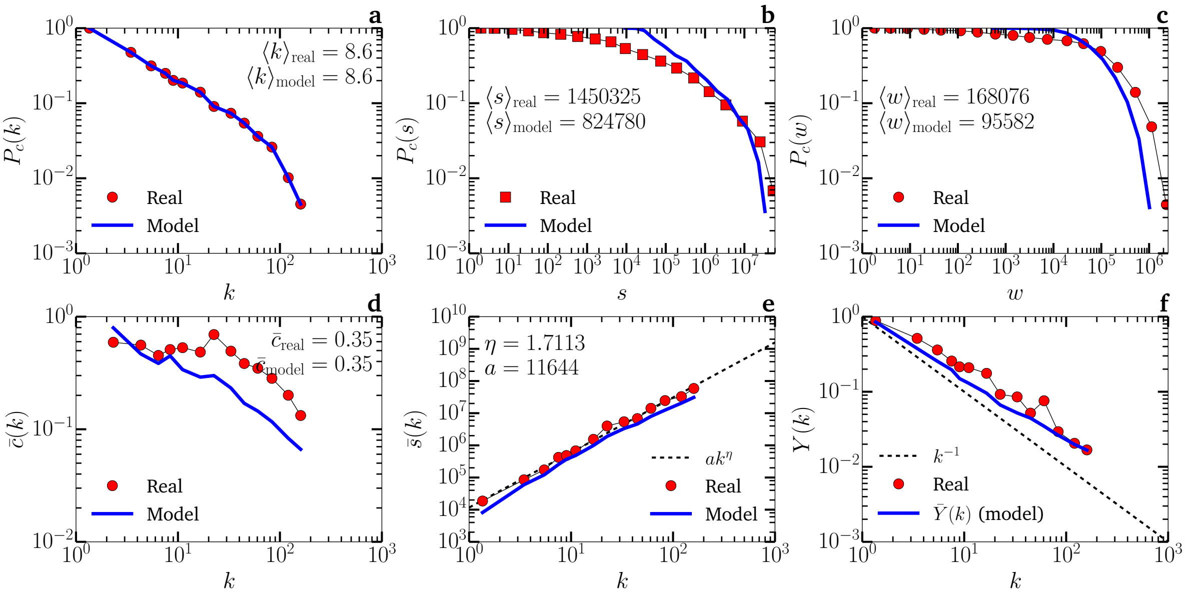

SUPPLEMENTARY FIGURE 28. **Predictions by Model C.** Comparison between topological and weighted properties of the US airports network (symbols) and a synthetic network generated by model C with $\mu = 1.05$ and $\nu = 0.225$ (solid lines). **a**, complementary cumulative degree distribution. **b**, complementary cumulative strength distribution. **c**, complementary cumulative weight distribution of links. **d**, degree-dependent clustering coefficient. **e**, average strength of nodes of degree k. **f**, disparity of nodes as a function of their degree.

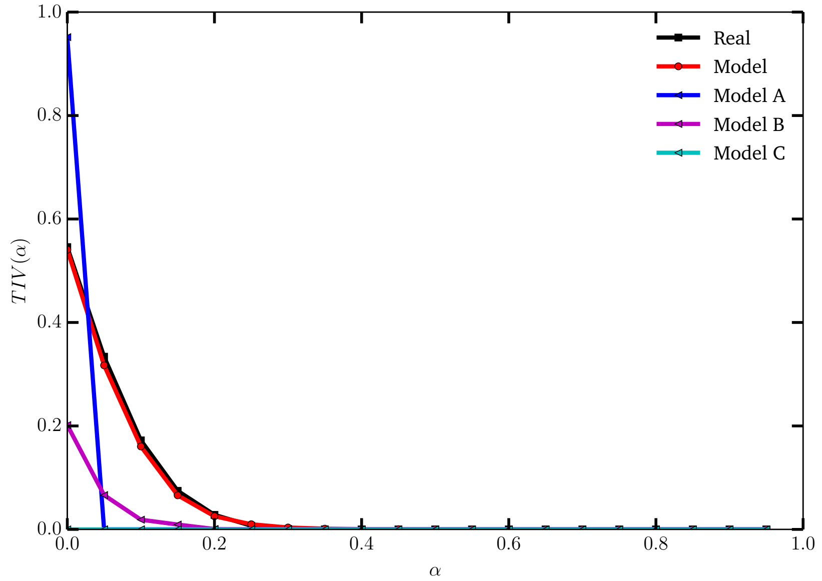

SUPPLEMENTARY FIGURE 29. **Triangle inequality violation spectrum.** Comparison between $TIV(\alpha)$ curves measured for the real US airports network, our model and the models A, B and C with the exponents given in the caption of Supplementary Figures 26–28.

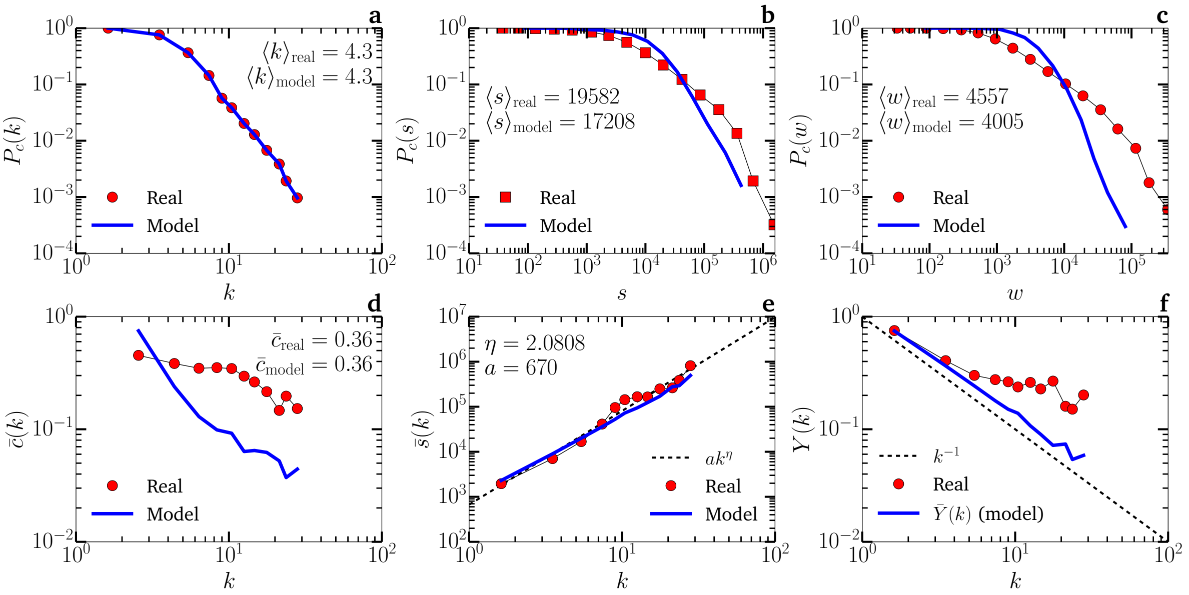

SUPPLEMENTARY FIGURE 30. **Predictions by Model A.** Comparison between topological and weighted properties of the US Commute network (symbols) and a synthetic network generated by model A with $\theta = 1.07$ (solid lines). **a**, complementary cumulative degree distribution. **b**, complementary cumulative strength distribution. **c**, complementary cumulative weight distribution of links. **d**, degree-dependent clustering coefficient. **e**, average strength of nodes of degree k. **f**, disparity of nodes as a function of their degree.

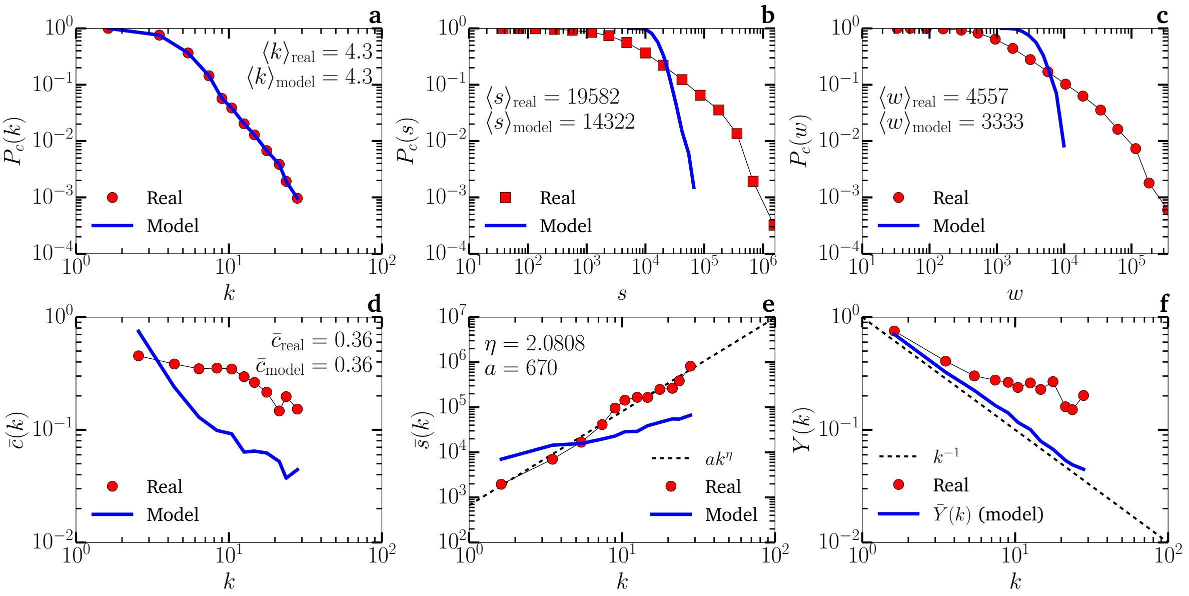

SUPPLEMENTARY FIGURE 31. **Predictions by Model B.** Comparison between topological and weighted properties of the US Commute network (symbols) and a synthetic network generated by model B with $\delta = 0.32$ (solid lines). **a**, complementary cumulative degree distribution. **b**, complementary cumulative strength distribution. **c**, complementary cumulative weight distribution of links. **d**, degree-dependent clustering coefficient. **e**, average strength of nodes of degree k. **f**, disparity of nodes as a function of their degree.

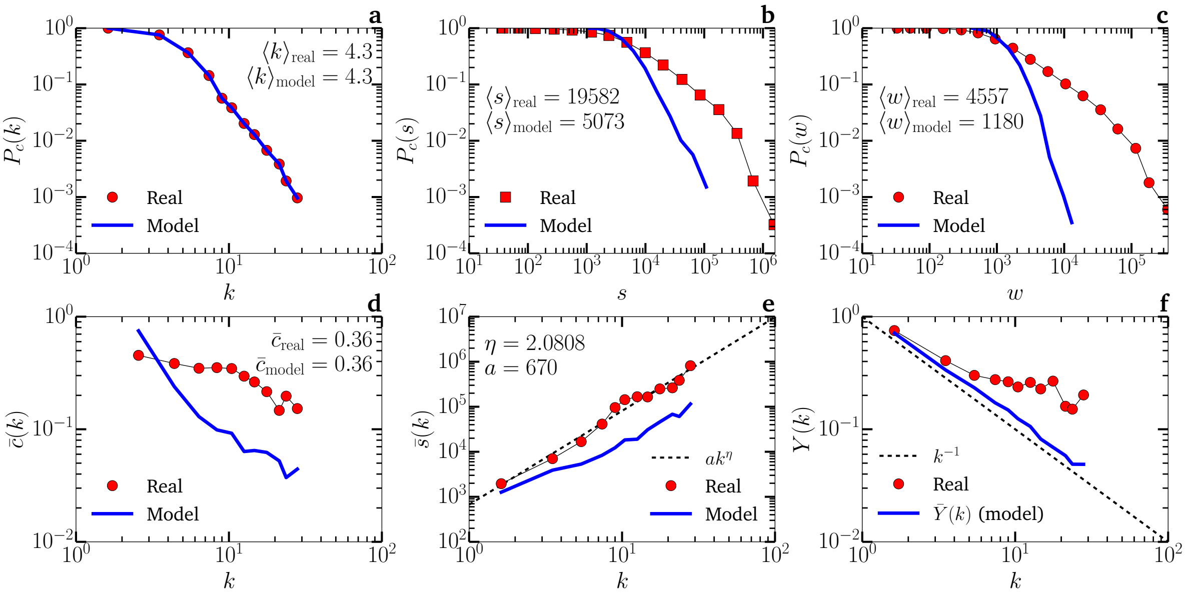

SUPPLEMENTARY FIGURE 32. **Predictions by Model C.** Comparison between topological and weighted properties of the US Commute network (symbols) and a synthetic network generated by model C with $\mu = 1.35$ and $\nu = 0.625$ (solid lines). **a**, complementary cumulative degree distribution. **b**, complementary cumulative strength distribution. **c**, complementary cumulative weight distribution of links. **d**, degree-dependent clustering coefficient. **e**, average strength of nodes of degree k. **f**, disparity of nodes as a function of their degree.

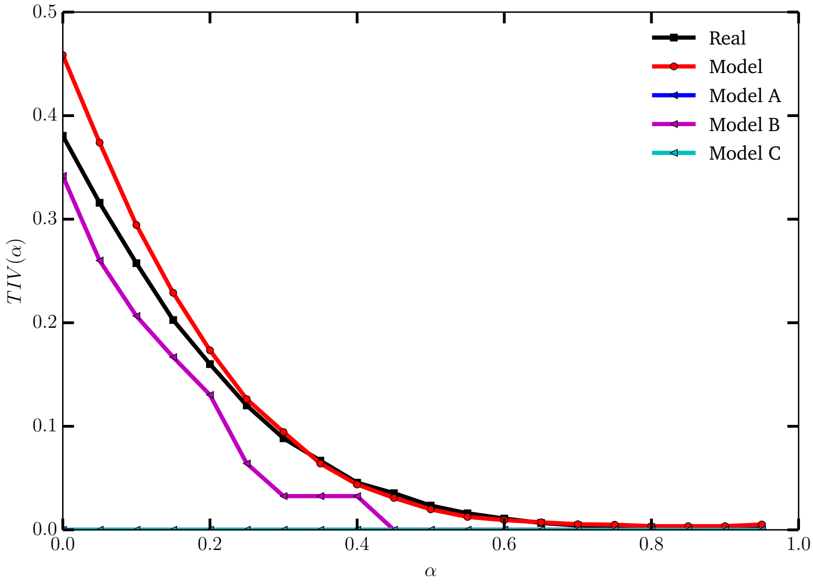

SUPPLEMENTARY FIGURE 33. **Triangle inequality violation spectrum.** Comparison between $TIV(\alpha)$ curves measured for the real US Commute network, our model and the models A, B and C with the exponents given in the caption of Supplementary Figures 30–32.

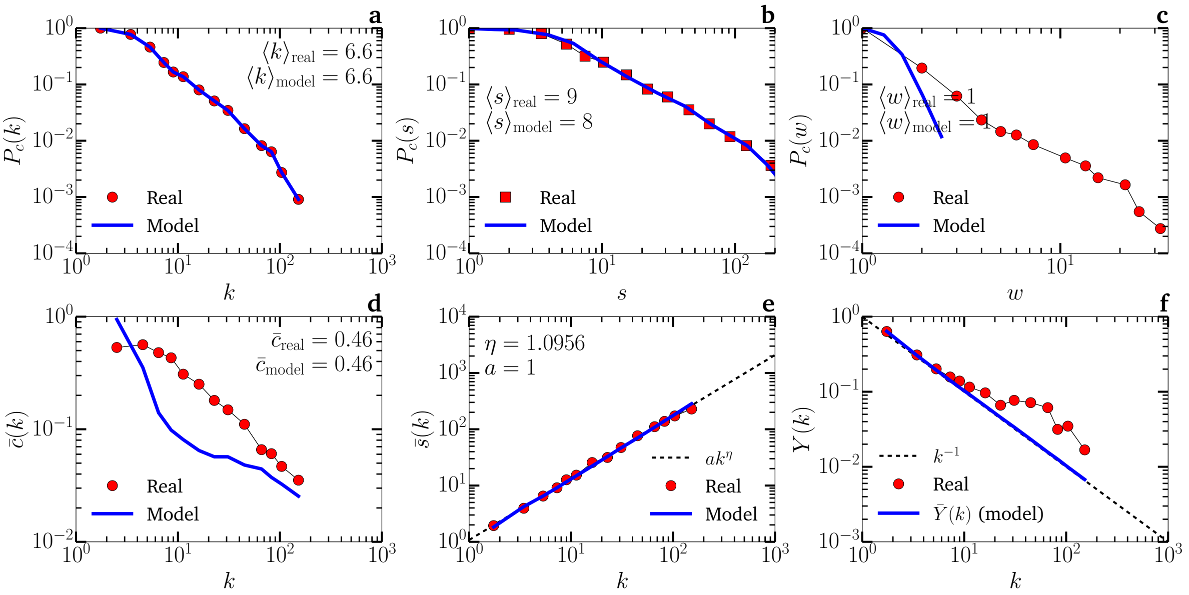

SUPPLEMENTARY FIGURE 34. **Predictions by Model A.** Comparison between topological and weighted properties of the iJO1366 *E. Coli* metabolic network (symbols) and a synthetic network generated by model A with $\theta = 0.16$ (solid lines). **a**, complementary cumulative degree distribution. **b**, complementary cumulative strength distribution. **c**, complementary cumulative weight distribution of links. **d**, degree-dependent clustering coefficient. **e**, average strength of nodes of degree k. **f**, disparity of nodes as a function of their degree.

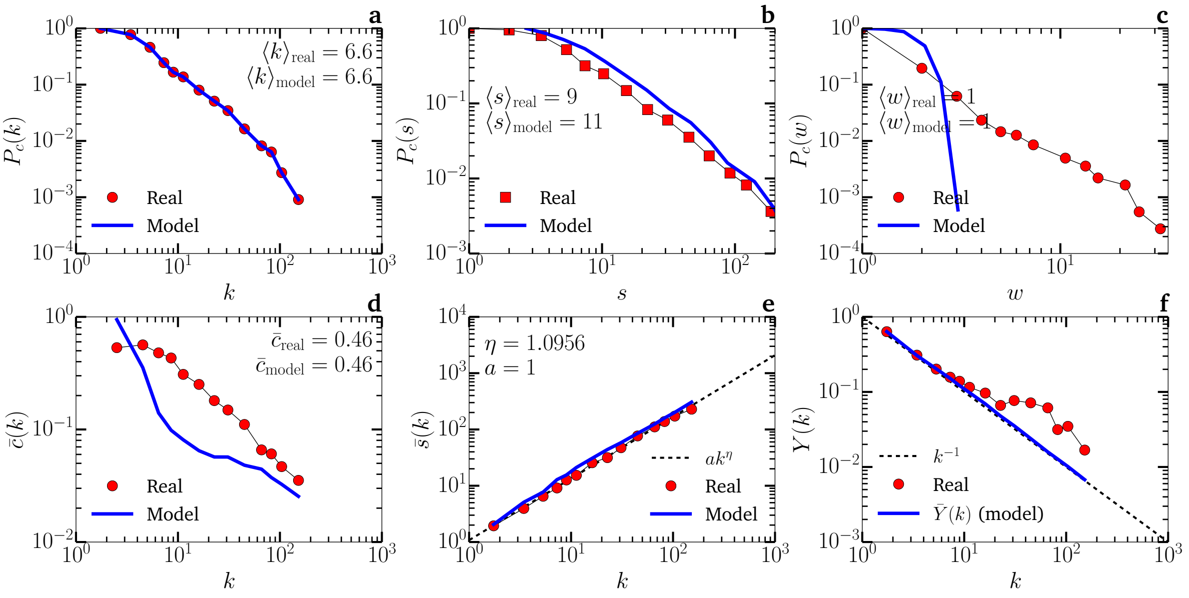

SUPPLEMENTARY FIGURE 35. **Predictions by Model B.** Comparison between topological and weighted properties of the iJO1366 *E. Coli* metabolic network (symbols) and a synthetic network generated by model B with $\delta = -0.15$ (solid lines). **a**, complementary cumulative degree distribution. **b**, complementary cumulative strength distribution. **c**, complementary cumulative weight distribution of links. **d**, degree-dependent clustering coefficient. **e**, average strength of nodes of degree k. **f**, disparity of nodes as a function of their degree.

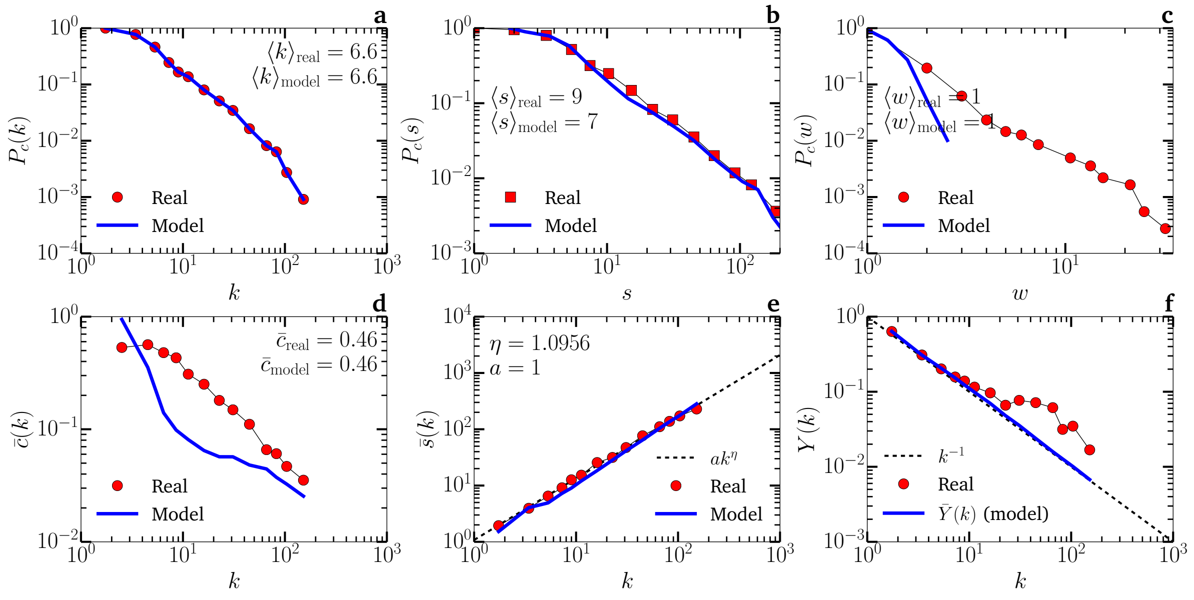

SUPPLEMENTARY FIGURE 36. **Predictions by Model C.** Comparison between topological and weighted properties of the iJO1366 *E. Coli* metabolic network (symbols) and a synthetic network generated by model C with $\mu = 0.225$ and $\nu = 0.075$ (solid lines). **a**, complementary cumulative degree distribution. **b**, complementary cumulative strength distribution. **c**, complementary cumulative weight distribution of links. **d**, degree-dependent clustering coefficient. **e**, average strength of nodes of degree k. **f**, disparity of nodes as a function of their degree.

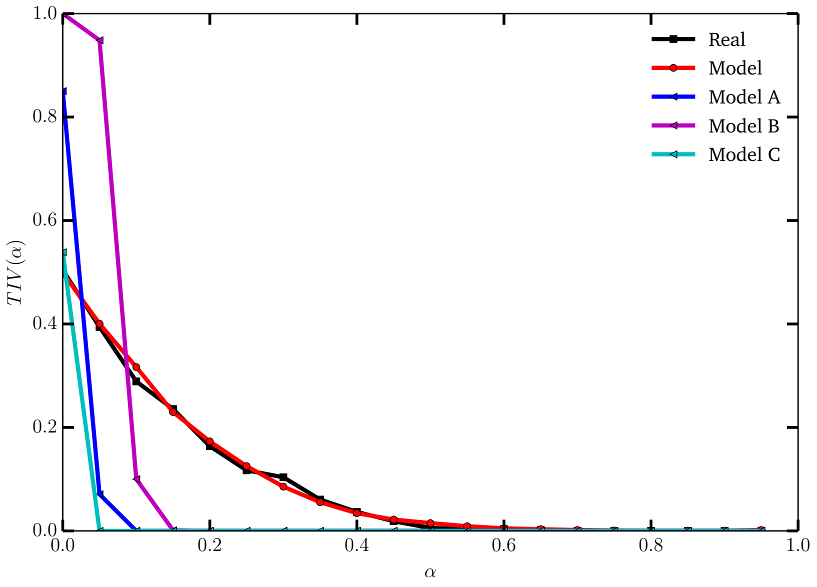

SUPPLEMENTARY FIGURE 37. **Triangle inequality violation spectrum.** Comparison between $TIV(\alpha)$ curves measured for the real iJO1366 *E. Coli* metabolic network, our model and the models A, B and C with the exponents given in the caption of Supplementary Figures 34–36.

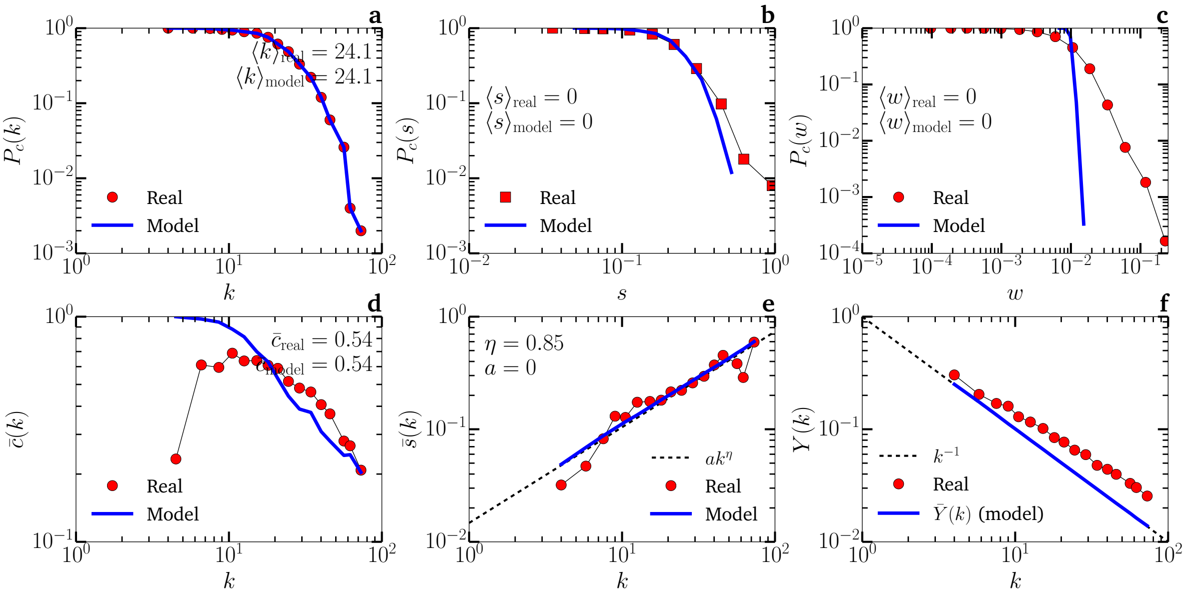

SUPPLEMENTARY FIGURE 38. **Predictions by Model A.** Comparison between topological and weighted properties of the Human brain network (symbols) and a synthetic network generated by model A with $\theta = -0.19$ (solid lines). **a**, complementary cumulative degree distribution. **b**, complementary cumulative strength distribution. **c**, complementary cumulative weight distribution of links. **d**, degree-dependent clustering coefficient. **e**, average strength of nodes of degree k. **f**, disparity of nodes as a function of their degree.

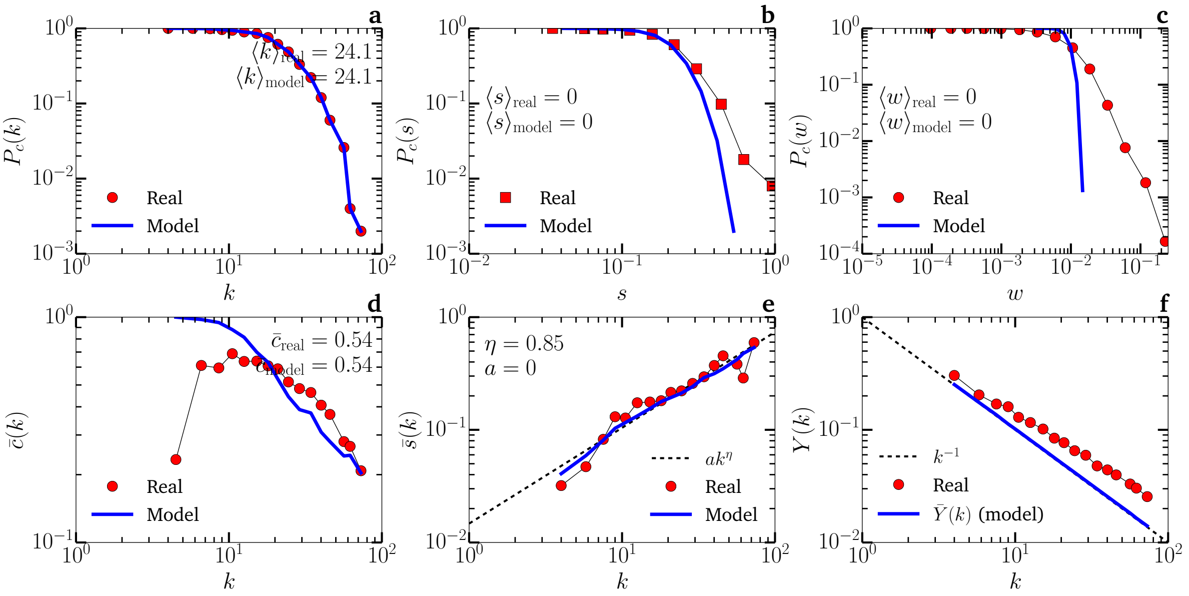

SUPPLEMENTARY FIGURE 39. **Predictions by Model B.** Comparison between topological and weighted properties of the Human brain network (symbols) and a synthetic network generated by model B with $\delta = 0.33$ (solid lines). **a**, complementary cumulative degree distribution. **b**, complementary cumulative strength distribution. **c**, complementary cumulative weight distribution of links. **d**, degree-dependent clustering coefficient. **e**, average strength of nodes of degree k. **f**, disparity of nodes as a function of their degree.

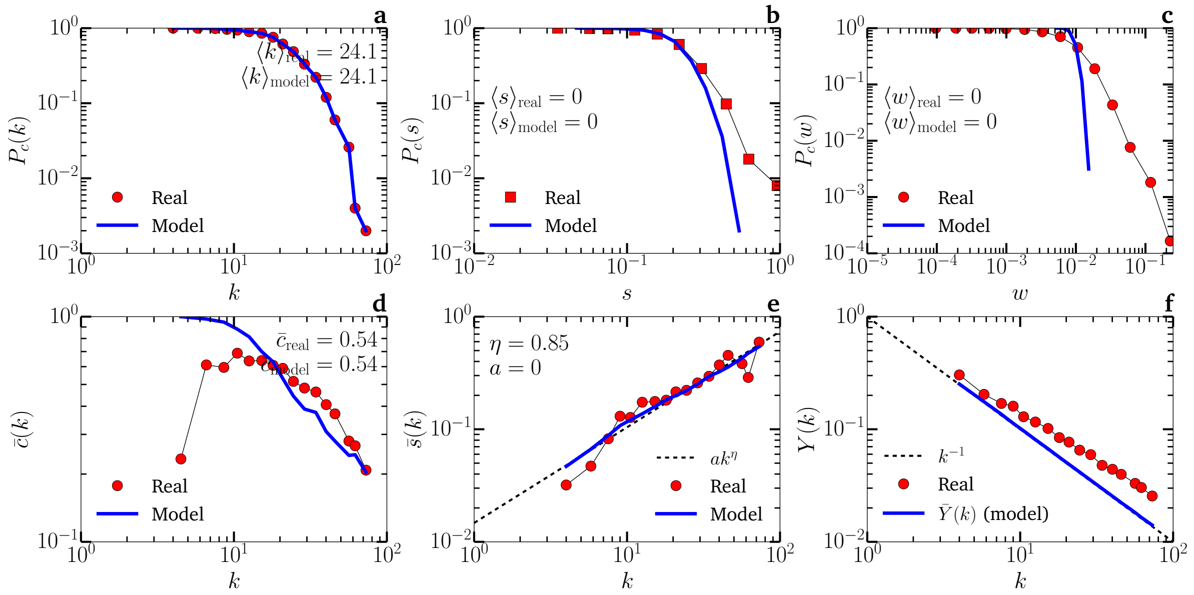

SUPPLEMENTARY FIGURE 40. **Predictions by Model C.** Comparison between topological and weighted properties of the Human brain network (symbols) and a synthetic network generated by model C with $\mu = -0.125$ and $\nu = 0.2$ (solid lines). **a**, complementary cumulative degree distribution. **b**, complementary cumulative strength distribution. **c**, complementary cumulative weight distribution of links. **d**, degree-dependent clustering coefficient. **e**, average strength of nodes of degree k. **f**, disparity of nodes as a function of their degree.

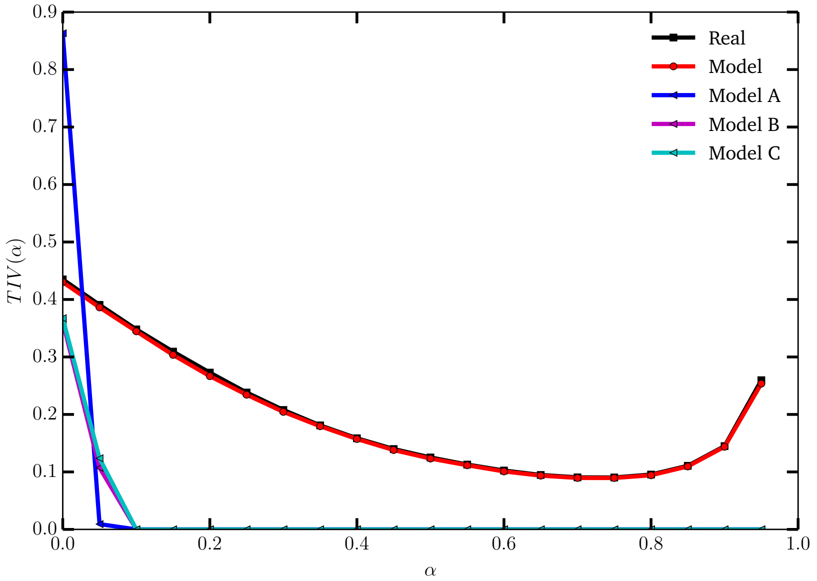

SUPPLEMENTARY FIGURE 41. **Triangle inequality violation spectrum.** Comparison between $TIV(\alpha)$ curves measured for the real Human brain network, our model and the models A, B and C with the exponents given in the caption of Supplementary Figures 38–40.

Supplementary References

[1] M. Á. Serrano, D. Krioukov, and M. Boguñá, "Self-similarity of complex networks and hidden metric spaces," Phys. Rev. Lett. **100**, 078701 (2008).

[2] M. Boguñá, D. Krioukov, and K. C. Claffy, "Navigability of complex networks," Nat. Phys. **5**, 74–80 (2009).

[3] M. Boguñá and D. Krioukov, "Navigating ultrasmall worlds in ultrashort time," Phys. Rev. Lett. **102**, 058701 (2009).

[4] M. Boguñá and R. Pastor-Satorras, "Class of correlated random networks with hidden variables," Phys. Rev. E **68**, 036112 (2003).

[5] D. Krioukov, F. Papadopoulos, M. Kitsak, A. Vahdat, and M. Boguñá, "Hyperbolic geometry of complex networks," Phys. Rev. E **82**, 036106 (2010).

[6] A. Barrat, M. Barthélemy, R. Pastor-Satorras, and A. Vespignani, "The architecture of complex weighted networks." Proc. Natl. Acad. Sci. U. S. A. **101**, 3747–52 (2004).

[7] F. W. J. Olver, D. W. Lozier, R. F. Boisvert, and C. W. Clark, *NIST Handbook of Mathematical Functions* (Cambridge University Press, New York, NY, 2010).

[8] S. N. Dorogovtsev and J. F. F. Mendes, "Evolution of networks," Adv. Phys. **51**, 1079–1187 (2002).

[9] M. Boguñá, R. Pastor-Satorras, and A. Vespignani, "Cut-offs and finite size effects in scale-free networks," Eur. Phys. J. B **38**, 205–209 (2004).

[10] M. Barthélemy, A. Barrat, R. Pastor-Satorras, and A. Vespignani, "Characterization and modeling of weighted networks," Physica A **346**, 34–43 (2005).

[11] M. Á. Serrano, M. Boguñá, and R. Pastor-Satorras, "Correlations in weighted networks," Phys. Rev. E **74**, 055101 (2006).

[12] S. Pajevic and D. Plenz, "The organization of strong links in complex networks," Nat. Phys. **8**, 429–436 (2012).

[13] C. Orsini, M. M. Dankulov, P. Colomer-de Simón, A. Jamakovic, P. Mahadevan, A. Vahdat, K. E. Bassler, Z. Toroczkai, M. Boguñá, G. Caldarelli, S. Fortunato, and D. Krioukov, "Quantifying randomness in real networks," Nat. Commun. **6**, 8627 (2015).

[14] P. Colomer-de Simón, M. Á. Serrano, M. G. Beiró, J. I. Alvarez-Hamelin, and M. Boguñá, "Deciphering the global organization of clustering in real complex networks." Sci. Rep. **3**, 2517 (2013).
